# Gapless Superconductivity From Extremely Dilute Magnetic Disorder in 2H‐NbSe_2‐*x*_S_*x*_

**DOI:** 10.1002/adma.202519118

**Published:** 2026-02-23

**Authors:** Jose Antonio Moreno, Mercè Roig, Víctor Barrena, Edwin Herrera, Alberto M. Ruiz, Samuel Mañas‐Valero, Antón Fente, Anita Smeets, Jazmín Aragón, Yanina Fasano, Beilun Wu, Maria N. Gastiasoro, Eugenio Coronado, José J. Baldoví, Brian M. Andersen, Isabel Guillamón, Hermann Suderow

**Affiliations:** ^1^ Laboratorio de Bajas Temperaturas y Altos Campos Magnéticos, Departamento de Física de la Materia Condensada, Instituto Nicolás Cabrera and Condensed Matter Physics Center (IFIMAC), Unidad Asociada UAM‐CSIC Universidad Autónoma de Madrid Madrid Spain; ^2^ Niels Bohr Institute University of Copenhagen Copenhagen Denmark; ^3^ Department of Physics University of Wisconsin‐Milwaukee Milwaukee Wisconsin USA; ^4^ Instituto de Ciencia Molecular (ICMol) Universidad de Valencia Paterna Spain; ^5^ MESA+ Institute for Nanotechnology University of Twente Enschede The Netherlands; ^6^ Low Temperatures Lab Centro Atómico Bariloche, CNEA Bariloche Argentina; ^7^ Donostia International Physics Center Donostia‐San Sebastian Spain

**Keywords:** disorder, gapless, magnetic impurities, STM, superconductivity, Yu–Shiba–Rusinov states

## Abstract

Most superconducting materials exhibit a vanishing density of states at the Fermi level and Anderson's theorem posits that the superconducting gap is robust against nonmagnetic disorder. Although dilute magnetic impurities lead to localized in‐gap states, these states typically have no bearing on the material's bulk superconducting properties. However, numerous experiments reveal a finite density of states at the Fermi level in systems with an apparently negligible number of magnetic impurities. Here, using scanning tunneling microscopy and self‐consistent Bogoliubov‐de Gennes calculations, we find that gapless superconductivity emerges in 2H‐${\mathrm{NbSe}}_{2-x}S$ 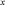 at remarkably low magnetic impurity concentrations. Furthermore, our density functional theory calculations and in‐gap quasiparticle interference measurements demonstrate that the Se‐S substitution significantly modifies the band structure. This modification favours nesting and dictates the in‐gap scattering for x>0, in stark contrast to the dominant charge density wave interactions in pure 2H‐${\mathrm{NbSe}}_{2}$. Our findings reveal an unusual superconducting response to disorder and highlight the importance of incorporating material‐specific band structures in the understanding of a superconductor's response to even very low concentrations of magnetic impurities.

## Graphical abstract

We demonstrate that 2H‐NbSe2‐xSx hosts gapless superconductivity at unexpectedly low magnetic impurity concentrations. Combining STM, Bogoliubovde Gennes simulations, DFT, and quasiparticle interference, we comprehensively study the development of gapless behavior and show that SeS substitution reshapes the band structure, enhances nesting, and drives in‐gap scattering. Our results reveal an unusual disorder response, emphasizing the decisive role of material‐specific electronic structure.

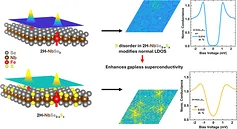

## 1 Introduction

Quantum materials are characterized by coupled quantum degrees of freedom which can give rise to a plethora of emergent ordered states, including, for example, superconductivity and charge density waves. Disorder can act as an important probe for such many‐body phases through the detailed response to local spatially modulated perturbations. In conventional s‐wave superconductors, nonmagnetic impurities generally do not significantly alter superconductivity due to the preservation of time‐reversed Kramer's pairs [1]. This explains the prevalence of superconductivity in numerous disordered metals [1, 2, 3, 4, 5, 6]. Despite this, a persistent observation in many superconductors – even those with seemingly negligible amounts of magnetic impurities – is a nonzero density of states (DOS) at the Fermi level [7, 8, 9, 10, 11, 12, 13, 14, 15]. Gapless superconductivity is often associated with magnetic order inside the superconducting phase [15, 16, 17], Bogoliubov Fermi surfaces [18, 19, 20], finite‐momentum superconductivity [21, 22] or Majorana bound states [23], and can emerge through a Lifshitz transition when induced by magnetic impurities [24, 25]. However, its connection to the normal phase electronic band structure and the electronic correlations of the host material is rarely explored. Although this relationship is fundamental in highly disordered and amorphous superconductors [2, 3, 4, 5, 6, 9, 12, 26, 27, 28], its relevance in single crystals with substitutional disorder remains unclear.

**FIGURE 1:**
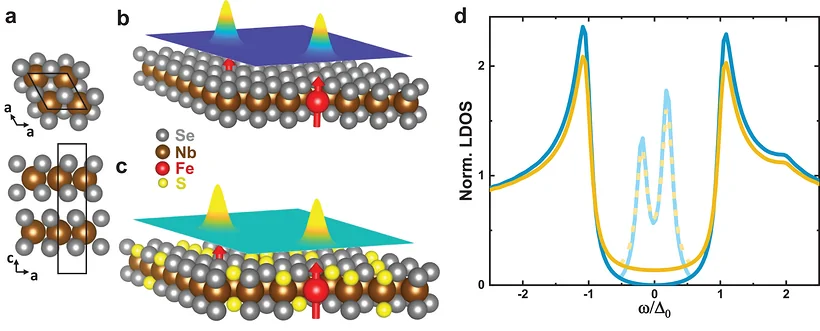
Emergence of gapless superconductivity in the 2H‐${\mathrm{NbSe}}_{2-x}S$ 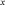 system with dilute magnetic impurities. (a) Schematic of the 2H‐${\mathrm{NbSe}}_{2}$ crystal structure: view of the aa‐plane (ac‐plane) in the top panel (bottom panel). The unit cell is indicated by the black line. Nb and Se atoms are brown and gray spheres, respectively. (b) Schematic showing a single Se‐Nb‐Se layer of 2H‐${\mathrm{NbSe}}_{2}$ containing two Fe magnetic impurities (red spheres with arrows). The color scale represents the local density of states (LDOS) at zero energy, from blue (zero LDOS) to yellow (LDOS peak at the impurity). (c) Similar to (b), but for a 2H‐${\mathrm{NbSe}}_{2-x}S$ 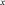 layer (x>0) with S substitutional disorder (yellow spheres). (d) Schematic representation of the LDOS as a function of energy ω normalized to the superconducting gap $\Delta_{0}$. Blue lines indicate pure 2H‐${\mathrm{NbSe}}_{2}$: dark (light) blue for LDOS far from (at) a magnetic impurity. Ocre lines represent 2H‐${\mathrm{NbSe}}_{2-x}S$ 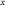 (x>0): dark (light) ocre for LDOS far from (at) an magnetic impurity. This panel highlights the modifications to the LDOS due to both Fe magnetic impurities and S substitution.

Here we use atomic‐scale scanning tunneling microscopy (STM) to investigate 2H‐${\mathrm{NbSe}}_{2-x}S$ 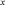 (0≤x≤0.8) doped with very low concentrations of randomly distributed Fe impurities (n≈ 0.02 at. %). We find that minute amounts of Fe disorder in conjunction with the S substitutional disorder cooperate to produce a gapless superconducting state. This is in stark contrast to the expectations from Abrikosov–Gorkov theory and early observations of magnetic pair breaking, in which gapless behavior requires magnetic impurity concentrations of the order of several at. % [29, 30, 31, 32]. By comparing experimental results with material‐specific theoretical simulations of in‐gap magnetic bound states and nonmagnetic disorder, we demonstrate that a comprehensive understanding of the resulting finite in‐gap DOS necessarily involves incorporating the changes in the normal state band structure induced by the nonmagnetic S substitution. In Figure 1, we provide a schematic illustration of the emergence of localized impurity states and gapless superconductivity from an interplay between such magnetic in‐gap bound states and substitutional disorder.

## 2 Results

**FIGURE 2:**
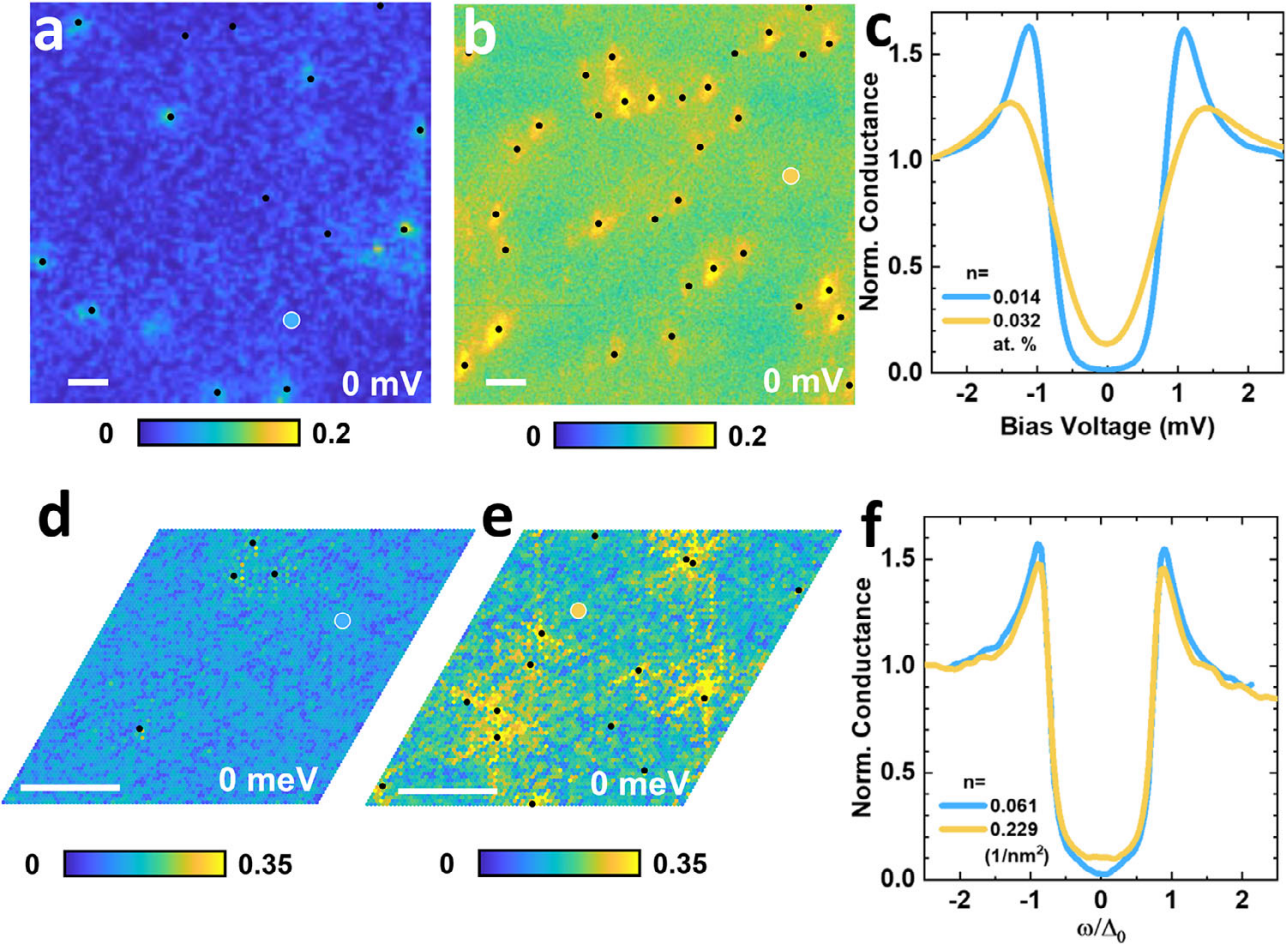
Gapless superconductivity in 2H‐${\mathrm{NbSe}}_{2-x}S$ 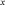 with magnetic impurities. (a,b) Zero bias tunneling conductance maps in 2H‐${\mathrm{NbSe}}_{1.8}S$ 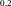 for low (a, n=0.014 at. %) and high Fe concentrations (b, n=0.032 at. %) obtained in different fields of view by STM. The temperature is $T/T_{c}\ll 0.05$. The color scale, ranging from zero (blue) to 0.2 (yellow) represents tunneling conductance normalized at bias voltages above the superconducting gap. Black dots mark the position of the Fe impurities. (c) Normalized tunneling conductance curves taken at locations far from impurities (locations marked with circles in a,b). (d,e) Calculated LDOS with 20% nonmagnetic impurity concentrations for low (d, n=0.061 at. %) and high Fe concentrations (e, n=0.229 at. %). Color scale ranges from zero (blue) to 0.35 (yellow) normalized LDOS. Black dots indicate the position of Fe impurities. (f) Calculated normalized LDOS curves averaged over (d,e). The curves are qualitatively similar to those taken far from impurities, for example at the locations marked with circles in (d,e). $\Delta_{0}$ is the energy of the superconducting gap. The white scale bars are 10 nm long in all maps.

Figure 2a–c displays tunneling conductance maps from two distinct regions of a 2H‐${\mathrm{NbSe}}_{1.8}S$ 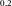 sample (we provide experimental details in Methods, Sections 5.1 and 5.2 and Table 1). The random distribution of Fe impurities, marked by black dots in Figure 2a,b, leads to substantial local variations of the impurity density. Despite the large inter‐impurity distances, well above the superconducting coherence length of ξ=7 nm in 2H‐${\mathrm{NbSe}}_{1.8}S$ 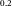, the tunneling conductance far from impurities consistently deviates from the flat and zero in‐gap conductance of clean BCS superconductors and exhibits instead a finite zero bias conductance, a continuous increase in the tunneling conductance vs bias voltage and suppressed quasiparticle peaks (Figure 2c). It is remarkable that such extremely dilute concentrations of introduced magnetic moments, corresponding to 1 moment per ∼3000 unit cells, are capable of producing strongly modified superconducting DOS throughout the field of view.

**TABLE 1:** Lattice constants and superconducting $T_{c}$ of single crystalline 2H‐${\mathrm{NbSe}}_{2-x}S$.

x in ${\mathrm{NbSe}}_{2-x}S$ 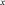	0	0.2	0.4	0.6	0.8
a=b (nm)	0.34451(3)	0.34327(5)	0.34197(3)	0.34099(4)	0.33984(4)
c (nm)	1.2542(1)	1.2506(2)	1.2474(2)	1.2423(2)	1.2387(2)
$T_{c}$(K)	7.2	6.2	5	4.6	3.3

**FIGURE 3:**
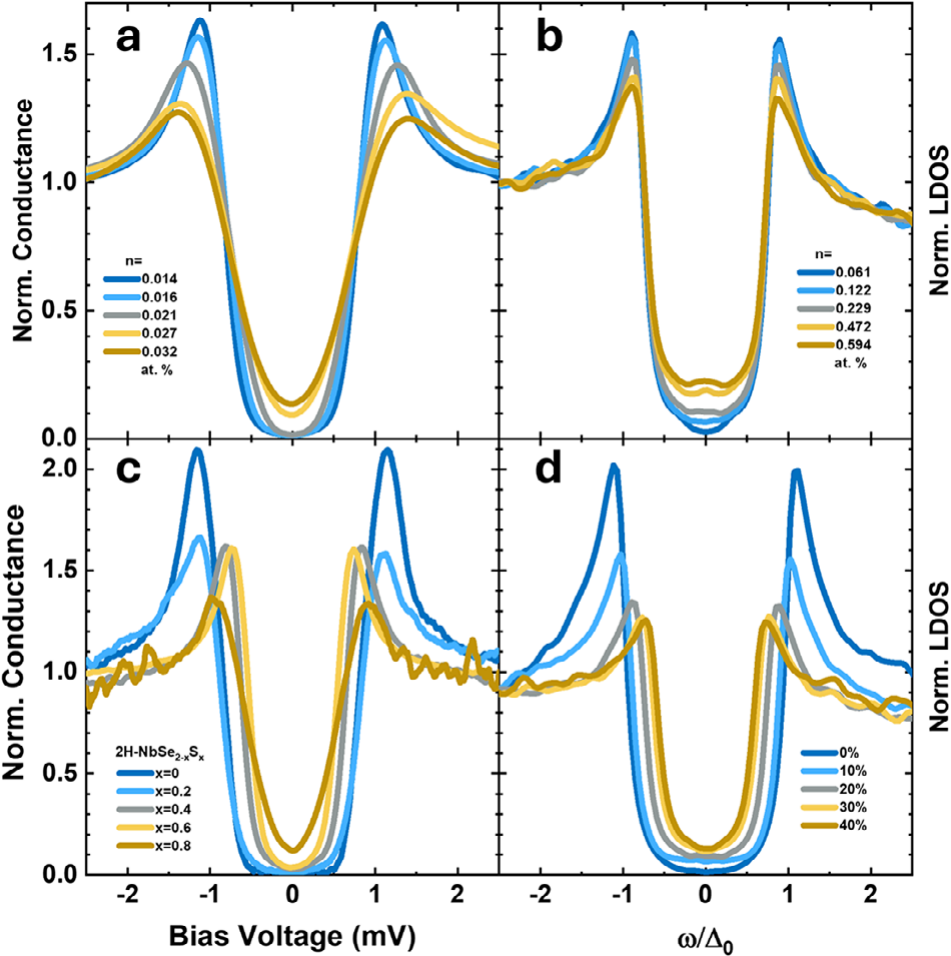
Local density of states as a function of magnetic and nonmagnetic impurity concentrations in 2H‐${\mathrm{NbSe}}_{2-x}S$ 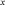. (a) Tunneling conductance as a function of the bias voltage at positions far from magnetic impurities (similar to the areas marked by blue and yellow dots in Figure 2a,b), and in different fields of view in 2H‐${\mathrm{NbSe}}_{1.8}S$ 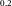 with different magnetic impurity concentrations n (n=0.014,0.016,0.021,0.027and0.032 at. % from blue to ocre). The conductance is normalized to its value for bias voltages above the superconducting gap. (b) Calculated normalized LDOS as a function of the energy far from magnetic impurities for different magnetic impurity concentrations (n=0.061,0.122,0.229,0.472and0.594 at. %, from blue to ocre) and with a 20% concentration of nonmagnetic impurity sites. (c) Tunneling conductance as a function of the bias voltage obtained at locations far from magnetic impurities and in fields of view with the smallest amount of impurities (about n≲0.02 %) in 2H‐${\mathrm{NbSe}}_{2-x}S$ 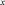 (x=0,0.2,0.4,0.6,0.8 from blue to ocre). We note that curves for x>0.2 present a finite conductance at zero voltage. Conductance values at zero voltage are shown in Figure S5. (d) Calculated normalized LDOS with concentration of sites with nonmagnetic impurities of 0%,10%,20%,30%,40% from blue to ocre for a magnetic impurity concentration of n=0.229%, except for the 0% nonmagnetic impurity case, which contains no magnetic impurities.

To understand these experimental findings, we present in Figure 2d,e the calculated local density of states (LDOS) maps obtained from self‐consistent Bogoliubov‐de Gennes simulations. These results are based on a microscopic model that includes randomly placed point‐like dilute magnetic moments on a triangular lattice with tight‐binding hopping parameters that reproduce the salient features of the band structure of 2H‐${\mathrm{NbSe}}_{2-x}S$ 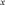 (all details are provided in Methods, Section 5.3 and Figures S1 and S2 of the Supporting Information). The obtained average LDOS shown in Figure 2f, which is representative of the typical LDOS curves in‐between magnetic impurities, successfully reproduces the experimentally observed enhanced LDOS close to the Fermi level. We return to a detailed discussion of the quantitative aspects of the comparison to experiments later. Our findings are consistent across multiple fields of views and for various 2H‐${\mathrm{NbSe}}_{2-x}S$ 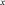 compositions (0.2≤x≤0.8). The experimental tunneling conductance far from magnetic impurities is presented in Figure 3a for 2H‐${\mathrm{NbSe}}_{1.8}S$ 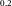 as a function of magnetic impurity concentration n; and in Figure 3c for 2H‐${\mathrm{NbSe}}_{2-x}S$ 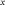 as a function of S content (x). The corresponding calculated LDOS are shown in Figure 3b,d. As seen, there is qualitative agreement with the experimental results, both in terms of magnetic and nonmagnetic disorder effects. First, as expected, the enhanced concentrations of magnetic moments pushes spectral weight into the superconducting gap, as seen from Figure 3a,b. Second, a key result is that increased substitutional nonmagnetic disorder clearly reduces the gap and amplifies the influence of magnetic impurities on the low‐energy LDOS, as seen from Figure 3c,d. For additional results, see Figures S2–S5 and, Sections S1–S3 and S5 in the Supporting Information. An understanding of the latter effect requires detailed knowledge of the general trends in the evolution of the normal state band structure with S substitution, an important topic to which we turn next.

**FIGURE 4:**
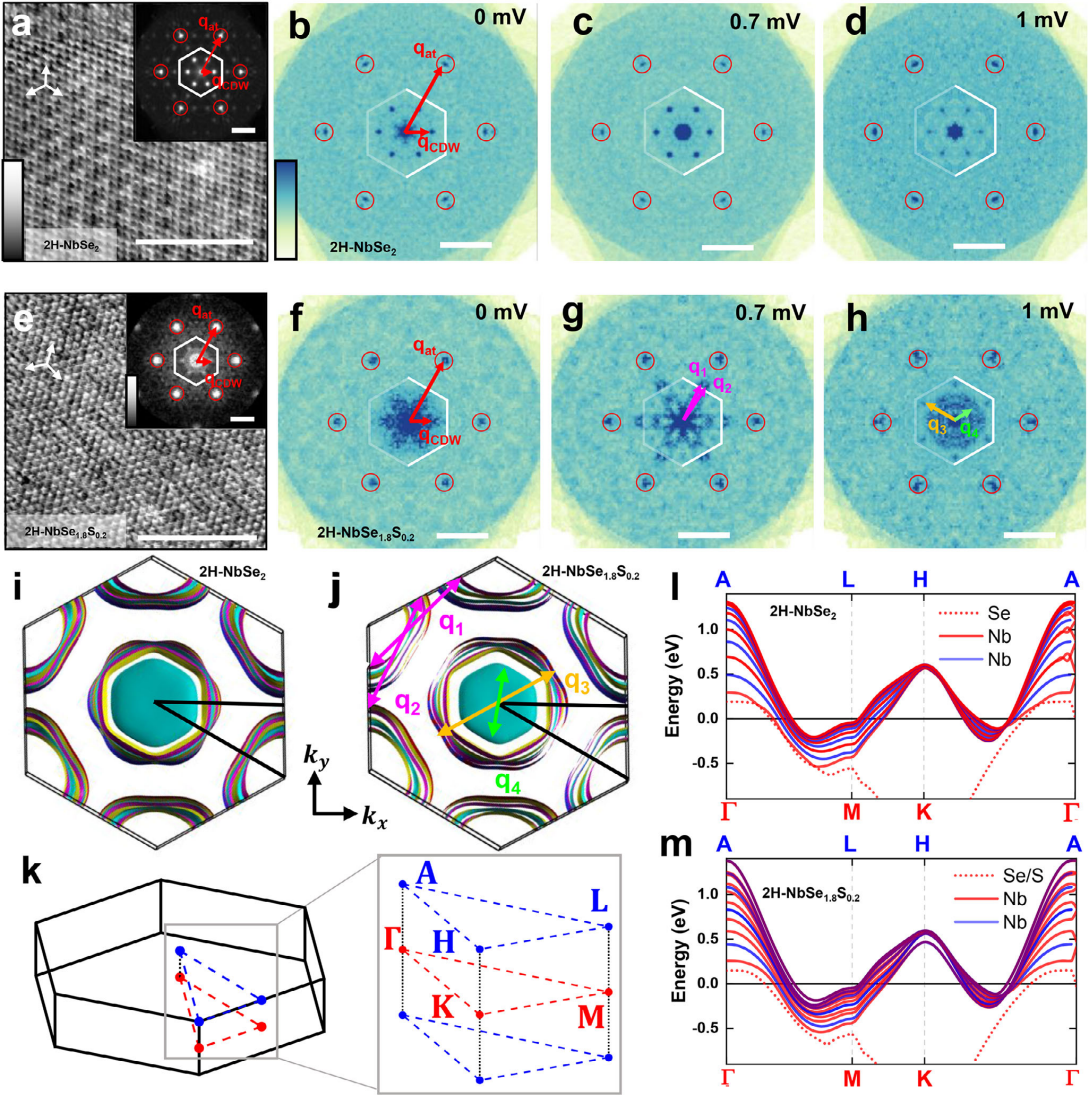
States at magnetic impurities and nesting. a (e) STM topography image obtained in 2H‐${\mathrm{NbSe}}_{2}$ (2H‐${\mathrm{NbSe}}_{1.8}S$ 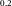). Insets show the respective Fourier transforms. White scale bars represent 5 nm in real space and 2 ${\mathrm{nm}}^{-1}$ in reciprocal space. White arrows indicate the direction of in‐plane crystal axes. White to black color scale corresponds to a height difference of 80 pm. (b–d) and (f–h) Fourier transform of tunneling conductance maps for three different bias voltages (b,f 0 mV, c,g 0.7 mV and d,h 1 mV) obtained in the same fields of view as a,e. The tunneling conductance maps for negative bias voltages are shown in Figure  S6 and provide the same results. The CDW $q_{CDW}$ and atomic lattice $q_{at}$ Bragg peaks are shown by red arrows. Bragg peaks are also surrounded by red circles. The white hexagons provide the boundaries of the first Brillouin zone. (i) (j) Fermi surface obtained from slab calculations of the band structures of 2H‐${\mathrm{NbSe}}_{2}$ (2H‐${\mathrm{NbSe}}_{1.8}S$ 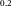). The Brillouin zone and the two main symmetry directions are marked by black lines. Colored arrows in (j) provide the different wave vectors identified in tunneling conductance images in (g,h). (k) Schematic representation of the first Brillouin zone of 2H‐${\mathrm{NbSe}}_{2-x}S$ 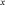. We show the main high symmetry points in the grey rectangle. l (m) Energy vs. wavevector dispersion relation in 2H‐${\mathrm{NbSe}}_{2}$ (2H‐${\mathrm{NbSe}}_{1.8}S$ 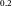). Red (blue) lines are bands derived from Nb orbitals along the Γ−M−K−Γ line (A‐L‐H‐A). We allow for mixed colors when two curves overlap. This occurs in the upper curves in (m), which are violet (red+blue) because the bands overlap, evidencing the absence of $k_{z}$ warping in the Fermi surface sheets for 2H‐${\mathrm{NbSe}}_{1.8}S$ 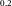. Dotted lines provide the Se/S derived band.

We now show that the scattering patterns produced inside the gap by magnetic impurities in 2H‐${\mathrm{NbSe}}_{2}$ and 2H‐${\mathrm{NbSe}}_{1.8}S$ 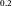 present compelling evidence for significant changes induced by S substitution in the electronic band structure. Tunneling conductance maps reveal spatial modulations of the LDOS, a characteristic feature of localized magnetic impurities. The wavelength of these modulations is dictated by the dominant features in the band structure responsible for pair‐breaking interaction [33, 34, 35, 36, 37]. Figure 4b–d show the Fourier transforms of these maps at selected in‐gap bias voltages for 2H‐${\mathrm{NbSe}}_{2}$. In contrast, Figure 4f–h displays the corresponding data for 2H‐${\mathrm{NbSe}}_{1.8}S$ 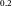 (topographies are shown in Figure 4a,e. Full data and real space maps are provided in Figure S6, see also Supporting Information, Section S3). In 2H‐${\mathrm{NbSe}}_{2}$, we observe LDOS modulations at reciprocal wave vectors corresponding to the atomic $q_{at}$ and charge density wave (CDW) $q_{CDW}$ periodicities, consistent with previous studies [34, 35, 36, 38, 39]. However, 2H‐${\mathrm{NbSe}}_{1.8}S$ 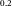 exhibits additional, distinct features. At bias voltages of 0.7 mV (Figure 4g), two new wave vectors, $q_{1}$ and $q_{2}$, emerge at the Brillouin zone boundary, clearly separated from the CDW wave vectors. Increasing the bias to 1 mV (Figure 4h) leads to a decrease in the intensity of these modulations, accompanied by the emergence of two additional wave vectors: $q_{3}$, rotated by 30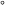 relative to the atomic and CDW lattices, and $q_{4}$, forming a near‐circular scattering pattern.

To understand the origin of the new in‐gap spatial oscillations, we performed density functional theory (DFT) calculations on two equivalent five‐unit‐cell slabs along the c‐axis for both 2H‐${\mathrm{NbSe}}_{2}$ and 2H‐${\mathrm{NbSe}}_{1.8}S$ 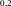 (see Methods Section 5.4 for details). The results, along with the 3D Brillouin zone, are presented in Figure  4i,j,l,m and Figure 4k, respectively. In pure 2H‐${\mathrm{NbSe}}_{2}$ (Figure 4i), the Fermi surface features a nearly spherical 3D Se‐derived pocket around the Γ− point of the Brillouin. The Nb‐derived tubular‐like Γ and M centered pockets show substantial $k_{z}$ warping. This difference in band dispersion between the Brillouin zone center (Γ−K−M) and at the upper basal (A−H−L) planes is clear in Figure 4l, where the corresponding energy dispersion curves do not overlap.

Upon S substitution in 2H‐${\mathrm{NbSe}}_{1.8}S$ 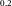 (Figure 4j), we observe a slight reduction in the size of the central Se/S‐derived pocket. More significantly, the Nb‐derived sub‐bands now exhibit well‐separated Fermi surface sheets within the 3D Brillouin zone. The crucial change occurs because S substitution substantially diminishes the $k_{z}$ warping. The varying S concentration for each plane within the slab effectively decouples the electronic band structure between layers, leading to sub‐bands with a pronounced 2D character. Consequently, the band dispersion in 2H‐${\mathrm{NbSe}}_{1.8}S$ 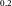 (Figure 4m) shows nearly identical dispersion at both the Brillouin zone center (Γ−K−M) and the upper basal (A−H−L) planes within each sub‐band.

We can correlate the wave vectors observed in the in‐gap scattering patterns (Figure 4g,h) with the features of the calculated band structures (Figure 4l,m). Specifically, the wave vector $q_{3}$ connects opposite, nearly flat portions of the hexagonal Nb‐derived Fermi surface pocket centered at Γ. This is consistent with the main nesting wave vector of the band structure [40]. Furthermore, the wave vector $q_{4}$ (green in Figure 4j) is related to the Se/S‐derived central pocket. Finally, the wave vectors $q_{1}$ and $q_{2}$ bridge the M and L points of the Brillouin zone, which are regions where the band structure exhibits significant energy curvature.

Consequently, S substitution in 2H‐${\mathrm{NbSe}}_{2-x}S$ 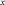 profoundly modifies the normal state electronic band structure compared to pure 2H‐${\mathrm{NbSe}}_{2}$. We note that $q_{1}-q_{4}$ are wave vectors that are also characteristic of the band structure of pure 2H‐${\mathrm{NbSe}}_{2}$. However, the c‐axis warping and the absence of non‐magnetic scattering centers reduce the scattering intensity associated with them in pure 2H‐${\mathrm{NbSe}}_{2}$. This in turn reduces the influence of the electronic features associated with the CDW in the quasiparticle scattering pattern of 2H‐${\mathrm{NbSe}}_{2-x}S$ 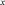. Instead, other electronic states distributed throughout the entire band structure now dominate the scattering. Concurrently, we observe a decrease in the superconducting critical temperature $T_{c}$ (Figure S7) and the suppression of the CDW with increasing sulfur concentration x (Figure  S8 and Section S4 in the Supporting Information).

## 3 Discussion

Although in‐gap Yu–Shiba–Rusinov (YSR) [41, 42, 43] states induced by magnetic impurities have been widely studied in pure 2H‐${\mathrm{NbSe}}_{2}$, these investigations consistently report a superconducting gap that remains fully open away from magnetic impurities [34, 36, 38, 39]. In contrast, gapless superconductivity in conventional systems typically emerges only at concentrations of magnetic impurities significantly higher than found here, on the order of several atomic percent as opposed to a fraction of a per mille [44, 45, 46, 47]. We stress that the gapless state found here appears despite the fact that for all x>0, the YSR bound states centered on magnetic impurities remain rather isolated and exhibit characteristics similar to those in 2H‐${\mathrm{NbSe}}_{2}$, including a pronounced electron‐hole asymmetry and an exponential decrease with distance (see Figures  S3–S5 and S9 and Sections S1– S3 and S5 in the Supporting Information).

Modifications of the normal state band structure are particularly important for understanding the x dependence of the LDOS in 2H‐${\mathrm{NbSe}}_{2-x}S$ 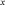 in Figure 3c,d. In the theoretical model, the normal state DOS at the Fermi level is reduced with nonmagnetic disorder. This has two important effects; first, it reduces the overall superconducting gap scale, in agreement with the narrowing of the coherence peaks seen both in experiment and calculations in Figure 3c,d. Second, the reduced DOS naturally enhances the tunneling conductance around zero bias, also in agreement with the results displayed in Figure 3c,d. This latter effect is discussed with more detail in Figure S2. Thus, in conclusion, the observed non‐zero LDOS at the Fermi level stems from the influence of nonmagnetic disorder in both the LDOS of magnetic YSR states, and the normal state LDOS.

There are also discrepancies between the experimental data and the theoretical findings. Our microscopic calculations require higher magnetic impurity concentrations to reproduce the experimental effects. For example, the experimental data in Figure 2b,c (0.032% Fe concentration) yields results comparable to calculations in Figure 2e,f, which correspond to a 0.229% magnetic impurity concentration. Despite this discrepancy, however, even this value is remarkably low for inducing gapless superconductivity [42]. The origin of the extreme sensitivity to magnetic impurities lies in the spatial extension of the YSR bound state wavefunctions. Their extent is often longer for reduced dimensionality, but also the normal state band structure can play an important role [48, 49, 50]. In the present case, for the clean system, the YSR states exhibit very long tails (see Figures  S1–S3), which can facilitate the finite LDOS far from impurities. Remarkably, even though the long YSR tails are smeared out by the nonmagnetic substitutional disorder (see Figure S3), the two types of disorder cooperate in promoting the gapless state as seen in Figure 2 and Figure S2. Furthermore, the experimentally observed quasiparticle peaks are more rounded and appear at higher bias voltage when introducing magnetic impurities, whereas they remain at roughly the same position in calculations. These discrepancies between theory and experiment suggest that our model may underestimate the cooperative influence of Se‐S substitutional disorder, or that there are additional relevant degrees of freedom, such as bond‐disorder, enhanced RKKY couplings from electronic correlations [51, 52, 53, 54, 55], variations in the electron‐phonon coupling, or electron‐electron interaction effects, maybe related to the new wave vectors $q_{1}-q_{4}$ observed in scattering (Figure 4).

We provide more details on the complex relationship between S substitution and CDW order in Figure S8 and Section S4 in the Supporting Information. Studies as a function of pressure in pure 2H‐${\mathrm{NbSe}}_{2}$ suggest that the CDW transition temperature should have a limited impact on the superconducting $T_{c}$ [56, 57, 58]. However, electron‐irradiated 2H‐${\mathrm{NbSe}}_{2}$ shows a concurrent suppression of $T_{c}$ and CDW [59]. Furthermore, London penetration experiments show that disorder provides a reduction of the in‐gap superconducting density of states related to the decrease of $T_{c}$, consistent with a disorder induced reduction of the superconducting gap anisotropy [59]. However, this is very different to the increased in‐gap density of states observed here. The non‐magnetic Se‐S substitutions play a key role in enhanced scattering. The level of disorder obtained by substitution is larger than the one obtained previously by irradiation. Nevertheless, in our case, there is still a periodic and coherent crystal lattice over the whole sample. Our data show that disorder plays a crucial role in modifying electronic interactions. It likely shifts the dominant interaction from a specific wave vector (the CDW wave vector), where the relevant interactions leading to the CDW are phonons [40, 60], toward electron–electron interactions. The latter involves band structure features, such as the nesting feature at $q_{3}$, which occur along different reciprocal space directions than in pure 2H‐${\mathrm{NbSe}}_{2}$. By investigating the CDW at local scale, we show that CDW occurs in patches, within very small length scales (just two or three in‐plane lattice constants, i.e. about a nm), and with exponentially decaying correlations (Figure S8 and Section S4). The crucial modifications in the band structure leading to these changes in the CDW could also influence superconductivity, favoring the appearance of a modified low energy LDOS.

## 4 Conclusion

In summary, our study reveals a continuous evolution toward gapless superconductivity in an s‐wave superconducting system, 2H‐${\mathrm{NbSe}}_{2-x}S$ 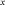. While material‐specific modeling that includes both magnetic and nonmagnetic disorder finds a very low concentration of magnetic moments required for inducing a gapless state, additional effects are necessary to quantitatively capture the observations. We have exposed how modifications in the normal state band structure plays an important role in facilitating an early onset of gapless superconductivity. The emergence of this state at remarkably low magnetic impurity concentrations highlights a cooperative interplay between Yu–Shiba–Rusinov bound states and nonmagnetic substitutional disorder on the resulting magnetic pair breaking effect in this superconducting material.

## 5 Methods

### 5.1 Sample Synthesis and Characterization

2H‐${\mathrm{NbSe}}_{2-x}S$ 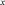 single crystals (x=0, 0.2, 0.4, 0.6, 0.8) of large sizes (above mm) were grown by a standard solid state reaction and a subsequent vapor transport process [61]. Crystals were doped with an Fe impurity concentration of ∼150 ppm for all samples (corresponding to n≈ 0.020 at. % accounting with experimental uncertainty). The Fe concentration was measured after the growth by inductively coupled plasma analysis. The concentration of other magnetic dopants was found to be <25 ppm using inductively coupled plasma spectroscopy.

In Table 1 we show the results of the macroscopic characterization of the samples. Elemental analysis from energy‐dispersive X‐ray spectroscopy (EDS) shows good agreement with the expected stoichiometry from the growth. By refining the powder x ray diffraction (XRD), we obtain the lattice parameters and find that there is a small reduction in both c and a lattice dimensions with increasing x. Diffraction peaks agree with the hexagonal 2H structure (space group P63/mmc). Superconducting parameters in Table 1 are obtained from resistivity.

### 5.2 Scanning Tunneling Microscopy

We used a home‐built STM thermally attached to a dilution refrigerator capable of resolving features in the tunneling conductance down to about 8 μV [62, 63]. We used Au tips, cleaned and prepared in situ as described in Ref. [64] and methods described in Ref. [65]. We use our own measurement and image treatment software, described in Ref. [66], complemented with other available software [67]. Clean atomically flat surfaces larger than 1 μm were obtained by cleaving the samples in situ by gluing an alumina piece to the surface and removing it at 4.2 K using a movable mechanically operated sample holder described in Ref. [68]. The exposed surfaces are Se/S hexagonal surfaces. Fourier transforms of real space maps have been symmetrized following the C6 symmetry of the surface lattice. It is important to stress that we make the measurements at temperatures far below the critical temperature $T_{c}$ ($<0.1T_{c}$), which varies in 2H‐${\mathrm{NbSe}}_{2-x}S$ 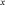 with x as shown in Table 1.

We have followed the tunneling conductance as a function of temperature in 2H‐${\mathrm{NbSe}}_{2-x}S$ 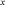 for x>0.2. The results are shown in Figure  S7. We can find the density of states at each temperature by deconvoluting the Fermi function and obtain the result shown in the right panels of Figure  S7a,b. From the density of states we find the superconducting gap as a function of temperature, obtaining a temperature dependence compatible with BCS theory (Figure S7c).

To estimate the local atomic concentration of magnetic impurities we mark the positions where we observe evidence for in‐gap states in the tunneling conductance vs bias voltage curves (similar to those shown in Figures S4d,h,l,p,t and S5g). These are shown as black dots in Figure 2 and Figures S4 and S5. We then count all black dots and divide by the number of Nb atoms, which are calculated by dividing the surface area of a given field of view with the area of a unit cell (there is one Nb atom per unit cell). We have analysed QPI data following Refs. [70, 71]. We have used the digital lock‐in technique described in Ref. [72]. and the autocorrelation function described in Ref. [12] to analyse CDW oscillations.

### 5.3 Numerical Modeling of the Superconducting LDOS

To analyze the effect of both magnetic and nonmagnetic impurities, we start from the standard BCS real space Hamiltonian given by,

(1) $$H=-\sum_{i,j,\sigma }t_{ij}c_{i\sigma }^{†}c_{j\sigma }-\mu \sum_{i,\sigma }c_{i\sigma }^{†}c_{i\sigma }+\sum_{i}\Delta_{i}\left(c_{i↑}^{†}c_{i↓}^{†}+H.c.\right)$$

where $c_{i,\sigma }^{†}$ ($c_{i\sigma }$) corresponds to the electronic creation (annihilation) operator, with i denoting the coordinates of a 2D triangular lattice and σ is the spin. For the kinetic term, we consider up to fifth nearest neighbor hoppings with the parameters $\left\{t_{1},t_{2},t_{3},t_{4},t_{5}\right\}=\left\{-23,-128.75,-2.2,7.5,-3\right\}$ and chemical potential μ=−275. All parameters considered in the numerical calculations are in units of meV. The crystalline structure contains two ${\mathrm{NbSe}}_{2}$ layers (Figure 1a), leading to two sets of Nb derived bands (Figure 4l,m). We assume that a single layer is sufficient to capture the main aspects of the in‐gap states. This is in line with our DFT calculations showing that the Se‐S substitutional disorder makes the bands more 2D.

The magnetic Fe impurities are implemented as:

(2) $$H_{m}=\sum_{i}\delta_{ir_{m}}V_{m}\left(c_{i↑}^{†}c_{i↑}-c_{i↓}^{†}c_{i↓}\right)$$

where $r_{m}$ corresponds to the positions of the magnetic impurities on the lattice. $V_{m}=75$ meV chosen such that the YSR states are located inside the superconducting gap at energies like the experimental observation. We note that superconductivity, following basic Abrikosov‐Gor'kov theory, is completely destroyed for scattering rates $2\pi DOS\left(E_{F}\right)V_{m}^{2}n$ larger than ≈Δ(T=0)/2. Here, $DOS(E_{F})$ is the normal state density of states at the Fermi level and Δ(T=0) denotes the order parameter at T=0 in the absence of disorder. This leads to destruction of superconductivity for magnetic impurity concentrations above n≈2−3%, which roughly agrees with experiments [29, 30, 31, 32]. Abrikosov–Gor'kov theory also predicts gapless superconductivity at roughly the same level of magnetic disorder, which is clearly in conflict with the gapless conductance measurements from extremely dilute magnetic disorder concentrations found in this work.

On the other hand, the Se‐S substitutional disorder is modeled by nonmagnetic impurities included as repulsive local potentials at random locations,

(3) $$H_{p}=V_{p}\sum_{i,\sigma }\delta_{ir_{p}}c_{i\sigma }^{†}c_{i\sigma }$$

with $r_{p}$ the lattice positions of the nonmagnetic potentials. We perform the calculations for fixed electron density and adjust the chemical potential accordingly. We choose $V_{p}=175$ meV so that the characteristic six‐fold shape of individual YSR states is washed out by disorder, as seen in the experiment (see Figure S3).

We construct the $2N^{2}\times 2N^{2}$ Bogoliubov‐de Gennes (BdG) Hamiltonian using the spinor $\Psi_{i}^{†}=\left(c_{i↑}^{†},c_{i↓}\right)$, with N denoting the system size in real space. We introduce the BdG transformation

(4) $$\left\{\begin{array}{c} c_{i\sigma }^{†}=\sum_{n=1}^{N^{2}}\left(u_{i\sigma }^{n^{*}}\gamma_{n}^{†}+v_{in}^{n}\gamma_{n}\right),\\ c_{i\sigma }=\sum_{n=1}^{N^{2}}\left(u_{i\sigma }^{n}\gamma_{n}+v_{i\sigma }^{n^{*}}\gamma_{n}^{†}\right) \end{array}\right.$$

where ${\left(u_{i↑}^{n},v_{i↓}^{n}\right)}^{⊤}$ denote the set of eigenvectors that diagonalize the BdG Hamiltonian with eigenvalues $E_{n}$, and hence the diagonalized Hamiltonian is given by $H=\sum_{n}E_{n}\gamma_{n}^{†}\gamma_{n}+E_{0}$. Here, the sum over n includes only the positive eigenenergies. Using the previous transformation, we can solve self‐consistently for the superconducting order parameter at each site,

(5) $$\Delta_{i}=V_{\mathrm{SC}}\left(\left\langle c_{i↑}c_{i↓}\right\rangle -\left\langle c_{i↓}c_{i↑}\right\rangle \right)$$

where $V_{\mathrm{SC}}$ is the on‐site attractive pairing interaction. In the numerical calculations, we choose $V_{\mathrm{SC}}=-90$ meV, which in the homogeneous case gives rise to a gap amplitude $|\Delta_{0}|∼5$ meV. We normalize the energy ℏω to $|\Delta_{0}|$ in the figures and we omit ℏ for clarity.

The site‐resolved LDOS for a frequency ω is obtained from the imaginary part of the retarded Green's function,

(6) $$N_{\sigma }\left(i,\omega \right)=-\frac{1}{\pi }ℑG_{\sigma }\left(i,\omega \right)$$

where $G_{\sigma }\left(i,\omega \right)$ is calculated from $G_{\sigma }\left(i,i\omega_{n}\right)=-\int_{0}^{\beta }d\tau e^{i\omega_{n}\tau }\left\langle T_{\tau }c_{i\sigma }\left(\tau \right)c_{i\sigma }\left(0\right)\right\rangle$ by taking $i\omega_{n}\to \omega +i\eta$, with η a small parameter that will determine the numerical resolution of the gap. Using the BdG transformation introduced in Equation (4), the total LDOS can be written as

(7) $$N\left(i,\omega \right)=-\frac{1}{\pi }ℑ\sum_{n,\sigma }\left(\frac{|u_{i\sigma }^{n}{|}^{2}}{\omega -E_{n}+i\eta }+\frac{|v_{i\sigma }^{n}{|}^{2}}{\omega +E_{n}+i\eta }\right)$$

We normalize the LDOS to its average from $\omega \approx -2\Delta_{0}$ to $\omega \approx -3\Delta_{0}$. We introduce a broadening η of approximately 2% $\Delta_{0}$ to account for the effects due to the finite size of the calculations. This leads, even for pure 2H‐${\mathrm{NbSe}}_{2}$, to a small finite value of the LDOS inside the gap. The blue curve in Figure S1a displays the spatially averaged LDOS for 2H‐${\mathrm{NbSe}}_{2}$. The ocre curve shows the LDOS at the site of a magnetic impurity. Figure S1b–d shows the real‐space dependence of the LDOS at ω=0 meV and at a bound state energy $\omega =\pm 0.2\Delta_{0}$. The LDOS around a magnetic impurity has a six‐fold shape in real space, which was discussed previously in Refs. [34, 49, 50].

The magnetic potential $V_{m}$ is fixed by the chosen YSR bound state energies in the single‐impurity case, see Figure S1a. We have performed extensive parameter‐dependence studies for varying $V_{p}$, magnetic disorder configurations, and also checked the dependence on μ. Overall, the results presented here are representative of all the performed studies. We note that the dependence of the spatially‐averaged LDOS vs. x shown in Figure 3d of the main text is not exhibited at all μ. Only band fillings where x itself causes sufficient changes (lowering) of the DOS near the Fermi level reproduce the experimental results in this respect. In Figure S2a, we show the non‐normalized DOS (alongside the corresponding normalized curves) for the cases of a distribution of nonmagnetic potential sites of 10%, 20%, 30% and 40%, with a magnetic impurity concentration of n=0.229%, $V_{m}=75$ meV and $V_{p}=175$ meV. From these plots, it is evident that the normal state DOS near the Fermi level is reduced upon increasing the concentration of nonmagnetic impurities. The origin of this effect can be traced to a smeared van Hove singularity in the simplified tight‐binding band.

In Figure S2b–d, we show the effect of increasing x in the zero energy density of states as a function of the position for an area without impurities Figure S2b, an area with one impurity Figure S2c, and an area with four impurities, Figure S2d. To visualize directly the effect of disorder in the zero energy density of states in the superconductor, the DOS is normalized to its value at energies well above the gap in Figure S2b–d. The effect of disorder on the zero energy DOS is pronounced already in absence of magnetic impurities. In the presence of magnetic impurities YSR states lose their characteristic six‐fold shape, and the increased DOS at zero energy is distributed more widely over the studied area.

We can also visualize that increasing the amount of disorder (Figure S3a–d) modifies the spatial extension of YSR states, distributing a finite density of states at the Fermi level over the whole area, and thus increasing the LDOS in between magnetic impurities. We obtain similar results when we make tunneling conductance maps at magnetic impurities (Figure S3i–k): Increasing the strength of disorder also suppresses the superconducting order parameter (Figure S3e–h), in agreement with the reduction of the gap amplitude shown in Figure 3d. We also note that in the pristine compound, the order parameter amplitude exhibits a slight decrease at the magnetic impurity sites (Figure S3e): With increasing disorder, this spatial variation is smeared out, resulting in only weak site‐dependent variations.

### 5.4 Density Functional Theory

Density Functional Theory (DFT) calculations were performed using the Quantum ESPRESSO package [69]. The exchange‐correlation energy was described using the Perdew–Burke–Ernzerhof (PBE) functional and the generalized gradient approximation (GGA). Van der Waals interactions between layers were included through the Grimme‐D3 dispersion correction scheme and pseudopotentials were extracted from the PSL library. During relaxation, we set a convergence threshold for total energy of 1× 10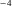 Ry, and 1× 10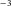 Ry/Bohr for the forces. The system studied is a 1×1×5 supercell of 2H‐${\mathrm{NbSe}}_{2}$, composed by 10 Nb and 20 Se atoms. To simulate the 2H‐${\mathrm{NbSe}}_{1.8}S$ 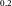 compound, two of the twenty Se atoms were substituted by S atoms. The two S atoms were placed at the maximum possible separation within the supercell (31.19 Å) to avoid artificial S–S interactions, thereby approximating the dilute and random nature of S substitution in experimental conditions. We employed an energy cutoff of 40 Ry for wavefunctions and 320 Ry for charge density and the Brillouin zone was sampled using a 16×16×3 Monkhorst–Pack k‐point mesh, centered at Γ, which ensures convergence of the total energy and electronic properties.

## Author Contributions

JAM and VB performed the experiments, with the supervision of EH, IG and HS. MR did the calculations with the supervision of MNG and BMA. Samples were synthesized and characterized by SMV and EC. DFT calculations were performed by AMR supervised by JJB. Vortex lattice experiments in doped samples were made by AF, JAM and JA, with the input of YF. VB and AS worked on the image treatment and interpretation. The study was devised by IG, HS, MNG and BMA. The paper was written by JAM, MR, AMR, JJB, MNG, BMA, IG and HS with input from all authors.

## Conflicts of Interest

The authors declare no conflicts of interest.

## Supporting information


**Supporting File**: adma72494‐sup‐0001‐SuppMat.pdf.

## Data Availability

The data generated in this study have been deposited in the osf.io database under accesion code https://doi.org/10.17605/OSF.IO/J8KDE.
