# Supplementary material for: Gapless Superconductivity From Extremely Dilute Magnetic Disorder in 2H‐NbSe2‐x S x
Source: Adv Mater. 2026 Feb 23;38(51):e19118. doi: 10.1002/adma.202519118 (PMC13568862; doi:10.1002/adma.202519118)
Supplement: Supplementary file 1 [file ADMA-38-e19118-s001.pdf]

Supporting Information for: Gapless superconductivity from extremely dilute magnetic disorder in 2H-NbSe$_{2-x}$S$_x$

Jose Antonio Moreno[1], Mercè Roig[2,3], Víctor Barrena[1], Edwin Herrera[1], Alberto M. Ruiz[4], Samuel Mañas-Valero[4], Antón Fente[1], Anita Smeets[1,5], Jazmín Aragón[6], Yanina Fasano[6], Beilun Wu[1], Maria N. Gastiasoro[7], Eugenio Coronado[4], José J. Baldoví[4], Brian M. Andersen[2], Isabel Guillamón[1], and Hermann Suderow[1,*]

[1]*Laboratorio de Bajas Temperaturas y Altos Campos Magnéticos, Departamento de Física de la Materia Condensada, Instituto Nicolás Cabrera and Condensed Matter Physics Center (IFIMAC), Unidad Asociada UAM-CSIC, Universidad Autónoma de Madrid, E-28049 Madrid, Spain*
[2]*Niels Bohr Institute, University of Copenhagen, DK-2100 Copenhagen, Denmark*
[3]*Department of Physics, University of Wisconsin–Milwaukee, Milwaukee, Wisconsin 53201, USA*
[4]*Instituto de Ciencia Molecular (ICMol), Universidad de Valencia, Catedrático José Beltrán 2, 49680, Paterna, Spain*
[5]*MESA+ Institute for Nanotechnology, University of Twente, 7500 AE, Enschede, The Netherlands*
[6]*Low Temperatures Lab, Centro Atómico Bariloche, CNEA, 8400 Bariloche, Argentina*
[7]*Donostia International Physics Center, 20018 Donostia-San Sebastian, Spain*

* Corresponding author: hermann.suderow@uam.es

S1. Electron-hole asymmetry of the in-gap magnetic states.

In Fig.S4 we show the tunneling conductance maps at different bias voltages in 2H-NbSe$_{1.8}$S$_{0.2}$ in fields of view of approximately 100 nm in lateral size for various local Fe impurity concentrations (14 Fe impurities, $n = 0.014$ %, Fig.S4**a-d**, 16 Fe impurities, $n = 0.016$ %, Fig.S4**e-h**, 22 Fe impurities, $n = 0.021$ %, Fig.S4**i-l**, 28 Fe impurities, $n = 0.027$ %, Fig.S4**m-p**, 33 Fe impurities, $n = 0.032$ %, Fig.S4**q-t**). The $+0.5$ mV conductance map with $n = 0.014\%$ (Fig.S4 **c**) shows brighter and more extended YSR states than its -0.5 mV counterpart (Fig.S4**a**). This situation is reversed for the field of view picked up with $n = 0.016\%$ where we observe a more pronounced hole character in Fig.S4**e** compared to Fig.S4**g**. As n increases, we observe regions with YSR states with both strong electron and hole features in Fig.S4**i,m,q** at -0.5 mV and Fig.S4**k,o,s** at $+0.5$ mV. The average difference between the density of states at positive and negative bias voltages within the gap and close to magnetic impurities remains of about 15% for all

concentrations of impurities n. Thus, the electron-hole asymmetry of YSR states remains unchanged as a function of magnetic impurity concentration and persists well within the range of gapless superconductivity.

S2. Gapless superconductivity and YSR states in 2H-$NbSe_{1.6}S_{0.4}$ and in 2H-$NbSe_{1.4}S_{0.6}$

In Fig.S5**a-c,d-f** we show tunneling conductance maps in 2H-$NbSe_{1.4}S_{0.6}$ and 2H-$NbSe_{1.6}S_{0.4}$, respectively, obtained in fields of view of similar size as those discussed in Fig.S4. We observe a very similar behavior of the YSR states to those described before in 2H-$NbSe_{1.8}S_{0.2}$ (Fig.S5**g**), with electron-hole asymmetric in-gap states and the emergence of gapless superconductivity with increasing x.

The average tunneling conductance at zero bias increases strongly with local impurity magnetic concentration and is larger for 2H-$NbSe_{2-x}S_x$ with $x > 0.2$ than for $x = 0.2$ (Fig.S5**h**, where we also show data from Fig.3**a**). Indeed, in 2H-$NbSe_{1.6}S_{0.4}$ we observe gapless superconductivity already for 0.01% Fe impurities. 2H-$NbSe_{1.4}S_{0.6}$ and 2H-$NbSe_{1.2}S_{0.8}$ show gapless superconductivity in all the fields of view that we have analyzed.

S3. In-gap states and electronic band structure in 2H-$NbSe_2$ and in 2H-$NbSe_{1.8}S_{0.2}$

In Fig.S6(**a-l**) we show tunneling conductance maps for several bias voltages in 2H-$NbSe_2$ and 2H-$NbSe_{1.8}S_{0.2}$ obtained in the field of view of Fig.4(**a,e**). The oscillatory behavior is seen on line scans (insets of Fig.S6**i-l**). These line scans provide the modulations that lead to the peaks observed in the Fourier transform shown in Fig.4. We provide the Fourier transforms for positive and negative bias voltages in Fig.S6(**m-x**).

The local density of states LDOS in reciprocal space and in presence of strong electron scattering is related to the band structure through $LDOS(E, \mathrm{q}) \propto |V_S(\mathrm{q})|^2 J(E, \mathrm{q})$, where E is the energy, $\mathrm{q} = \mathrm{k}_1 - \mathrm{k}_2$ the scattering wave vectors k_1 and k_2 are joining band structure constant energy contours (at E, we consider elastic scattering), V_S the scattering potential, here by Se-S

substitutional defects and $J(E, \mathrm{q})$ the joint density of states, $J(E, \mathrm{q}) = N_1(E, \mathrm{k}_1)N_2(E, \mathrm{k}_2)$.[1,2] The CDW wave vector in pure 2H-$NbSe_2$ is the main scattering wave vector, with a joint density of states $J(E, \mathrm{q})$ enhanced by electron-phonon interaction .[3] In 2H-$NbSe_{1.8}S_{0.2}$, by contrast, we observe the wave vectors $q_1 - q_4$. Their corresponding joint density of states $J(E, \mathrm{q})$ is much larger than elsewhere in the band structure, evidencing the shift in the wave vectors of the relevant interactions in presence of S disorder. The features commented in the main text and found in calculations are the sources of the enhanced joint density of states at these wave vectors. These are the van Hove anomaly close to the M point (q_1, q_2) and the pronounced two-dimensional character of the band structure in 2H-$NbSe_{1.8}S_{0.2}$ with a reduction of c-axis warping improving nesting on parallel Fermi surface sheets (q_3, q_4).

S4. Charge density wave in 2H-$NbSe_{2-x}S_x$

We show the evolution of the CDW peaks in 2H-$NbSe_{2-x}S_x$ in Fig.S8. We observe an exponential suppression of the Bragg peaks of the CDW with increasing x (Fig.S8**f**).

Real space maps as those shown in Fig.S8 can be written in the form

$$T(\mathbf{r}) = \sum_{n=1}^{3} A_{CDW,n}(\mathbf{r})\, e^{i\,\mathbf{q}_{CDW,n}\cdot\mathbf{r}} + ..., \tag{1}$$

where $\mathbf{r} = (x, y)$ is a real space vector, $\mathbf{q}_{CDW,n}$ are the three CDW wave vectors, and $A_{CDW,n}$ the amplitude of the oscillations of the CDW .[4] All other contributions to the maps are added to the first term of Eq. 1. We can now isolate the term $A_{CDW,n}$ by multiplying by $e^{i\,\mathbf{q}_{CDW,n}}$, shifting in reciprocal space the origin to the nth peak of the CDW, and multiplying by a Gaussian filter. We get

$$A_{CDW,n}(\mathbf{r}) = \frac{1}{\sqrt{2\pi}\sigma} \int d\mathbf{R}\, T(\mathbf{R})\, e^{i\,\mathbf{q}_{CDW,n}\cdot\mathbf{R}}\, e^{\frac{(\mathbf{r}-\mathbf{R})}{2\sigma_r^2}}, \tag{2}$$

where σ_r is the cutoff length of the Gaussian filter in real space. σ_r is large to include all the information of the CDW signal, but small enough to exclude other patterns. We compute $A_{CDW,n}$

in reciprocal space and then Fourier transform back to real space,

$$A_{CDW,n}(\mathbf{r}) = F^{-1}[A_{CDW,n}(\mathbf{q})] = \\ = F^{-1}\left[F\left(T(\mathbf{r})\, e^{i\,\mathbf{q}_{CDW,n}\cdot\mathbf{r}}\right)\frac{1}{\sqrt{2\pi}\sigma}e^{\frac{\mathbf{q}^2}{2\sigma_q^2}}\right], \tag{3}$$

with $\sigma_q = 1/\sigma_r$ and $F[\,]$ representing the Fourier transform.

Now, we can trace $T_{CDW,n}(\mathbf{r}) = A_{CDW,n}(\mathbf{r})\, e^{i\,\mathbf{q}_{CDW,n}\cdot\mathbf{r}}$ and obtain maps of the spatial-dependent of the amplitude of the CDW, $|A_{CDW,n}(\mathbf{r})|$. We then calculate the radially-averaged autocorrelation function $ACF(r)$ [5] and show the result in Fig.S8**g-i**.

Whereas the $ACF(r)$ in 2H-$NbSe_{1.8}S_{0.2}$ and in 2H-$NbSe_{1.6}S_{0.4}$ present an exponential decay, in 2H-$NbSe_2$ it shows a power law decay. Thus, we conclude that the long-range coherence CDW order present in 2H-$NbSe_2$ is suppressed by S substitutional disorder, where CDW only occurs within very small nanometer-sized patches.

S5. Spatial dependence of the in-gap states at magnetic impurities.

In Fig.S9**a,b** we show maps of the zero bias density of states in 2H-$NbSe_{1.8}S_{0.2}$ and in 2H-$NbSe_{1.6}S_{0.6}$. In Fig.S9**c,d** we show the radially-averaged LDOS around magnetic impurities. We find an exponentially decaying LDOS $\propto e^{-r/r_0}/r\, + C$. The term C accounts for the gapless background. We find r_0 of order of a few nm in all cases, showing that magnetic in-gap states are essentially independent from each other.

S6. Vortex lattice.

In Fig.S10**a-k** we show the superconducting vortex lattice for 2H-$NbSe_{2-x}S_x$, $x \leq 0.8$ for magnetic fields applied parallel to the c-axis. We observe in all cases a hexagonal vortex lattice, with the inter-vortex distance following the magnetic field dependence from an Abrikosov triangular lattice (Fig.S10**l**). We thus see that the substitutional disorder does not influence the vortex lattice symmetry.

We analyze the vortex cores in Fig.S10(**m-r**). The vortex core bound states characteristic of 2H-$NbSe_2$ are notably suppressed in 2H-$NbSe_{2-x}S_x$ for $x > 0.2$. A small peak in the tunneling conductance at the center of the vortex core remains for x≤0.6.

In Ref. ,[6] it was shown that the electron-hole asymmetry in the density of states characteristic of the YSR states is transferred to the vortex core bound states. As a consequence, the tunneling conductance maps of vortex cores are electron-hole asymmetric. This provides spatially asymmetric maps when subtracting the tunneling conductance σ at positive from the one at negative bias, $\sigma(+V) - \sigma(-V)$. As we show in Fig.S10**q,r** the electron-hole asymmetry of the vortex cores remains in 2H-$NbSe_{2-x}S_x$ for $x > 0.2$, in agreement with the magnetic states maintaining their electron-hole asymmetry for $x > 0.2$ (Fig.S5**a-f**).

References

[1] Fente, Antón, Meier, William R., Kong, Tai, Kogan, Vladimir G., Bud'ko, Sergey L.,Canfield, Paul C.,Guillamón, Isabel, & Suderow, Hermann Influence of multiband sign-changing superconductivity on vortex cores and vortex pinning in stoichiometric high-T_c $\mathrm{CaKFe_4As_4}$. *Phys. Rev. B* **97**, 134501 (2018).

[2] Hoffman, J. E., McElroy, K., Lee, D.-H., Lang, K. M., Eisaki, H.,Uchida, S., & Davis, J. C. Imaging Quasiparticle Interference in $Bi_2Sr_2CaCu_2O_{8+\delta}$. *Science* **297**, 1148-1151 (2002).

[3] Johannes, M. D., Mazin, I. I. & Howells, C. A. Fermi-surface nesting and the origin of the charge-density wave in $NbSe_2$. *Phys. Rev. B* **73**, 205102 (2006).

[4] Lawler, M. *et al.* Intra-unit-cell electronic nematicity of the high-T_c copper-oxide pseudogap states. *Nature* **466**, 347–351 (2010).

[5] Postolova, S. V. *et al.* Superconductivity in a disordered metal with Coulomb interactions. *Phys. Rev. Research* **2**, 033307 (2020).

[6] Park, S. *et al.* Coherent coupling between vortex bound states and magnetic impurities in 2D layered superconductors. *Nature Communications* **12**, 4668 (2021).

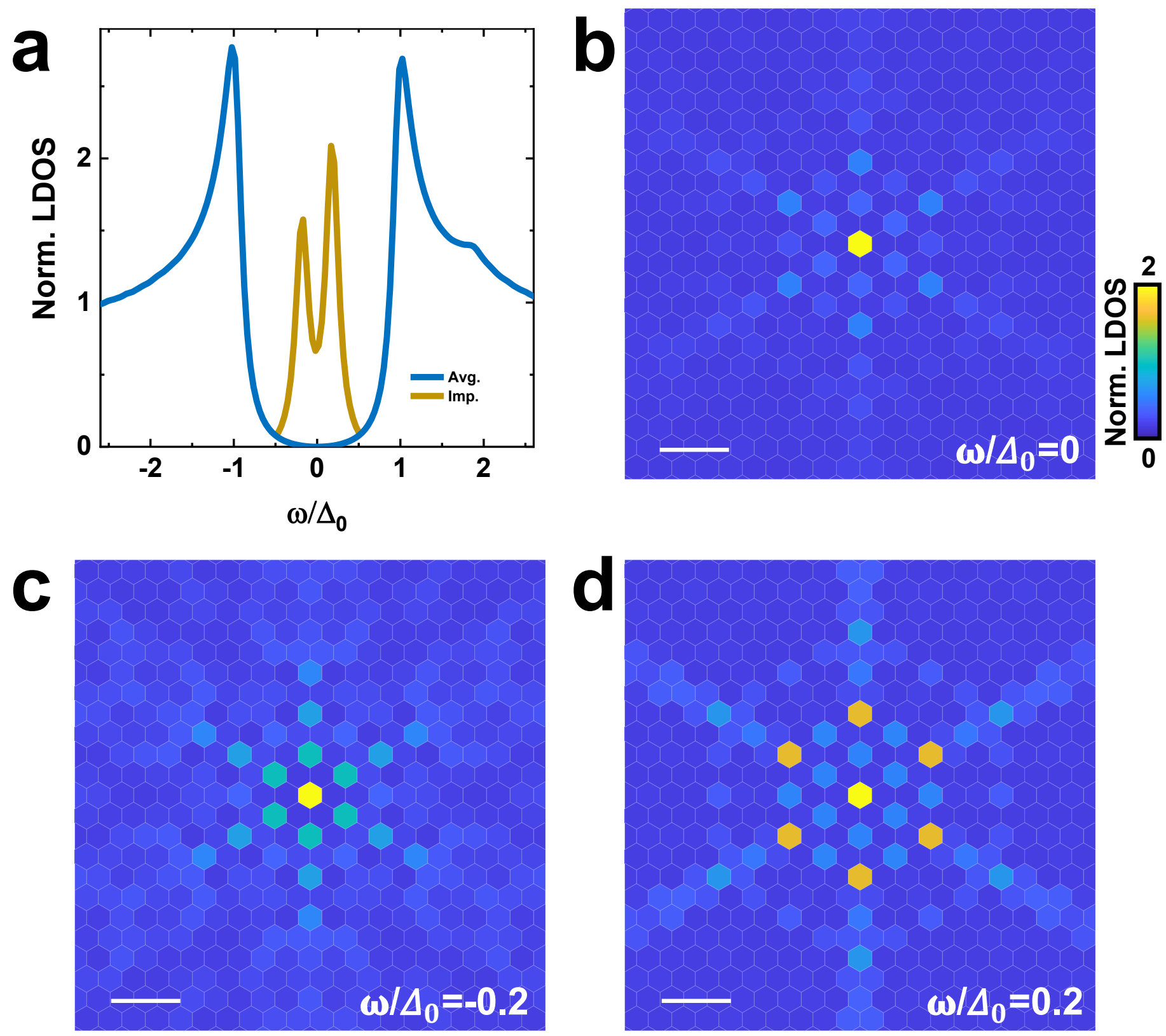

Figure S1 Calculated LDOS around a magnetic impurity. a The calculated LDOS in absence of magnetic impurities is shown as a blue curve and on top of a single magnetic impurity as an ocre curve (we use $V_m = 75$ meV). The systems is 81×81 sites in size. **b-d** Calculated maps of the LDOS with an impurity located exactly at the center. The LDOS follows the color scale shown in the lateral bar, from large (yellow) to small (blue). White scale bars represent 1 nm **b** is at the Fermi level and **c,d** are obtained at the bound state energies $\omega = \pm 0.2\Delta_0$ (peaks in the ocre curve in **a**).

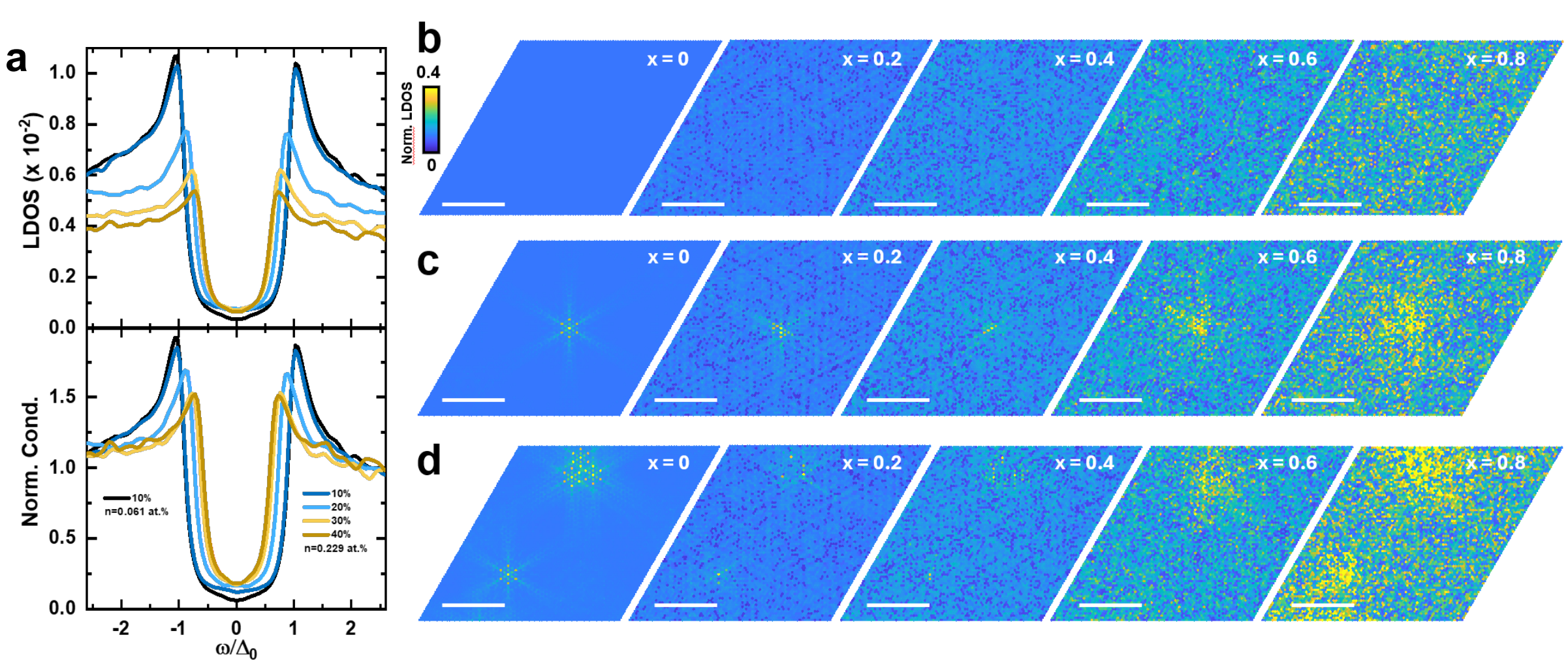

Figure S2 Non-normalized and normalized local density of states for different concentrations of nonmagnetic potential sites. (**a**) Top (bottom): Calculated non-normalized (normalized) LDOS as a function of energy with nonmagnetic potential sites of $10\,\%, 20\,\%, 30\,\%, 40\,\%$ from blue to ocre, for a magnetic impurity concentration $n = 0.229\%$. Black line represents $10\,\%$ nonmagnetic potential sites with $n = 0.061\%$. A reduction of the normal state DOS and a suppression of the superconducting gap value with x is evident. **b-d** Site-resolved normalized LDOS with several non-magnetic disorder configurations with no magnetic impurities (**b**, we note that disorder strongly modifies the density of states of the normal state), 1 magnetic impurity (**c**) and 4 magnetic impurities (**d**). The color bar is shown at the left. For all calculations the system size is of 81×81 sites. We use $V_m = 75$ meV, $V_p = 175$ meV, and $\omega = 0$. White scale bars represent 10 nm.

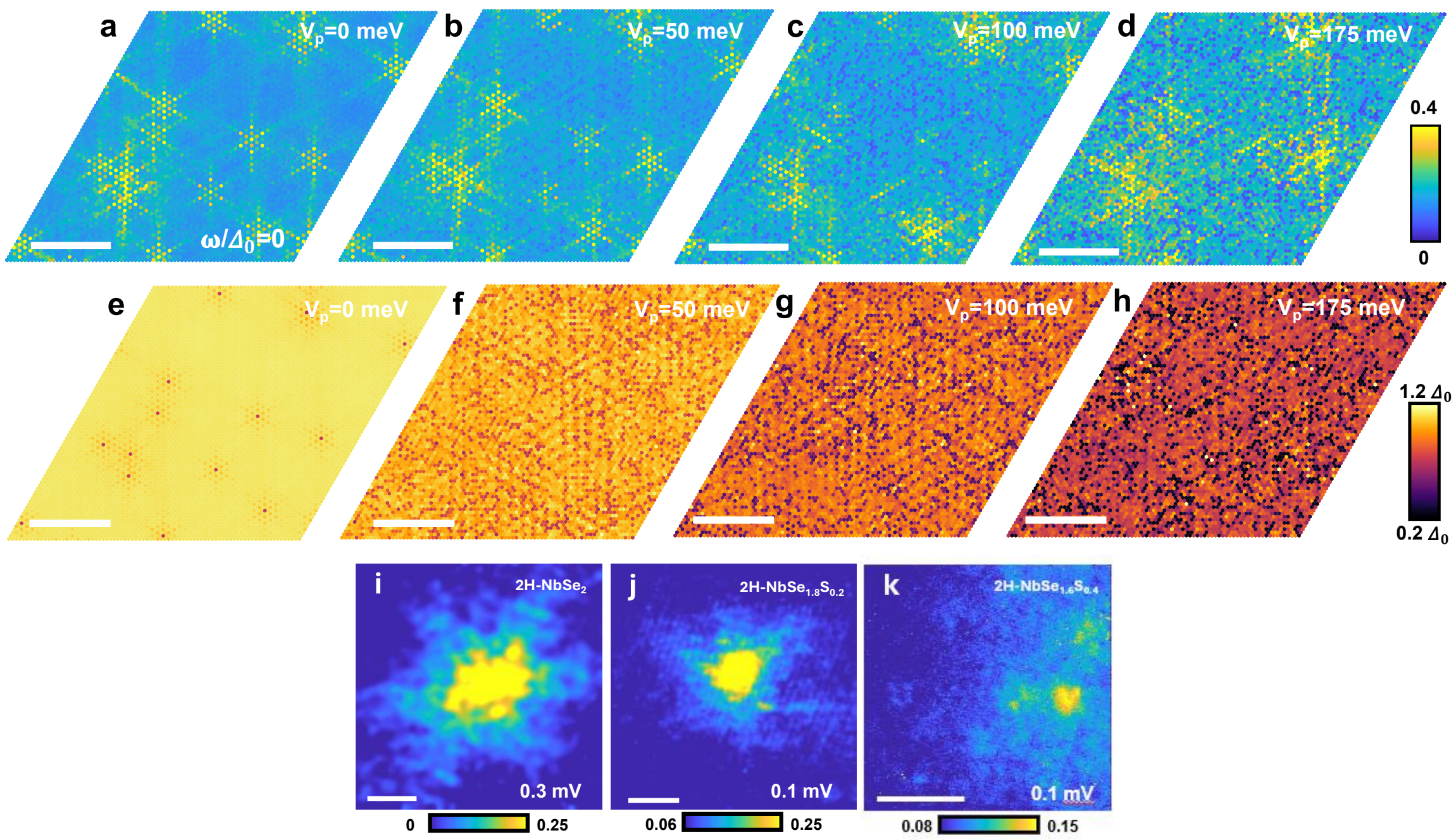

Figure S3 Influence of non-magnetic disorder on in-gap states and superconducting order parameter. Site-resolved normalized LDOS with nonmagnetic disorder, considering a magnetic impurity concentration $n = 0.061\%$ and a concentration of nonmagnetic impurity sites of $20\,\%$ in **b-d**. **e-h** Site-resolved superconducting order parameter Δ_0 corresponding to the maps shown in **a-d**. The system size is of 81×81 unit cells, $V_m = 75$ meV and **a,e** $V_p = 0$ meV, **b,f** $V_p = 50$ meV, **c,g** $V_p = 100$ meV and **d,h** $V_p = 175$ meV. White scale bar represents 10 nm. **i-k** Tunneling conductance maps at isolated magnetic impurities for **i** 2H-$NbSe_2$, **j** 2H-$NbSe_{1.8}S_{0.2}$, and **k** 2H-$NbSe_{1.6}S_{0.4}$. White scale bar is 2 nm long.

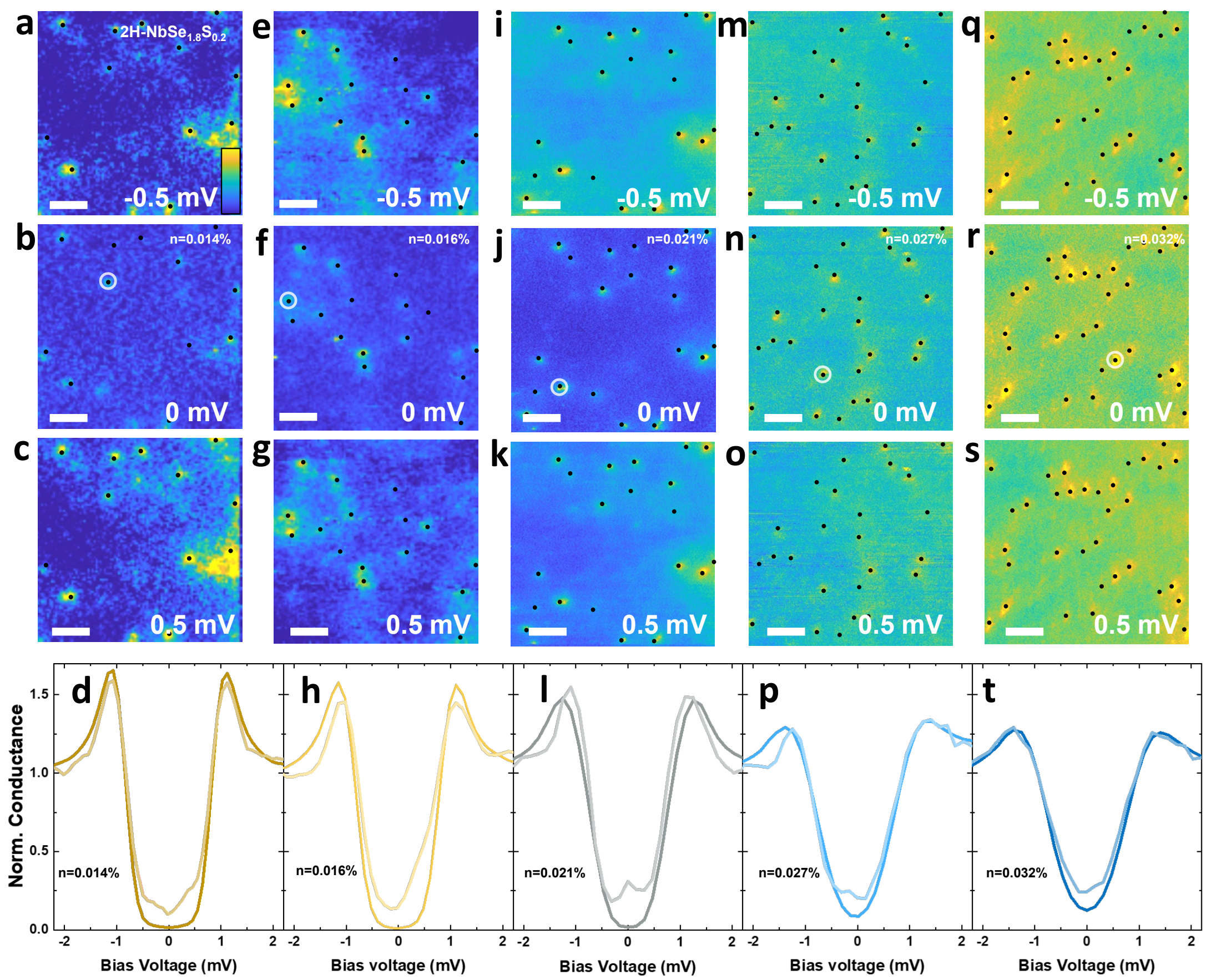

Figure S4 Tunneling conductance maps at different magnetic impurity concentrations. Tunneling conductance maps made in 2H-NbSe$_{1.8}$S$_{0.2}$ in fields of view with different n, at -0.5 mV (**a,e,i,m,q**), 0 mV (**b,f,j,n,r**) and $+0.5$ mV (**c,g,k,o,s**). Black dots provide the positions of Fe impurities. The tunneling conductance vs bias voltage curves at the impurities marked by a white circle is shown as bright colored lines in **d,h,l,p,t**. Curves away from impurities are also shown as shaded colored lines. The local magnetic impurity concentration n is 0.014% in **a-d**, 0.016% in **e-h**, 0.021% in **i-l**, 0.027% in **m-p** and 0.032% in **q-t**. White scale bars represent 20 nm. The conductance follows the color bar shown in **a**, with yellow being large and blue small values of the conductance.

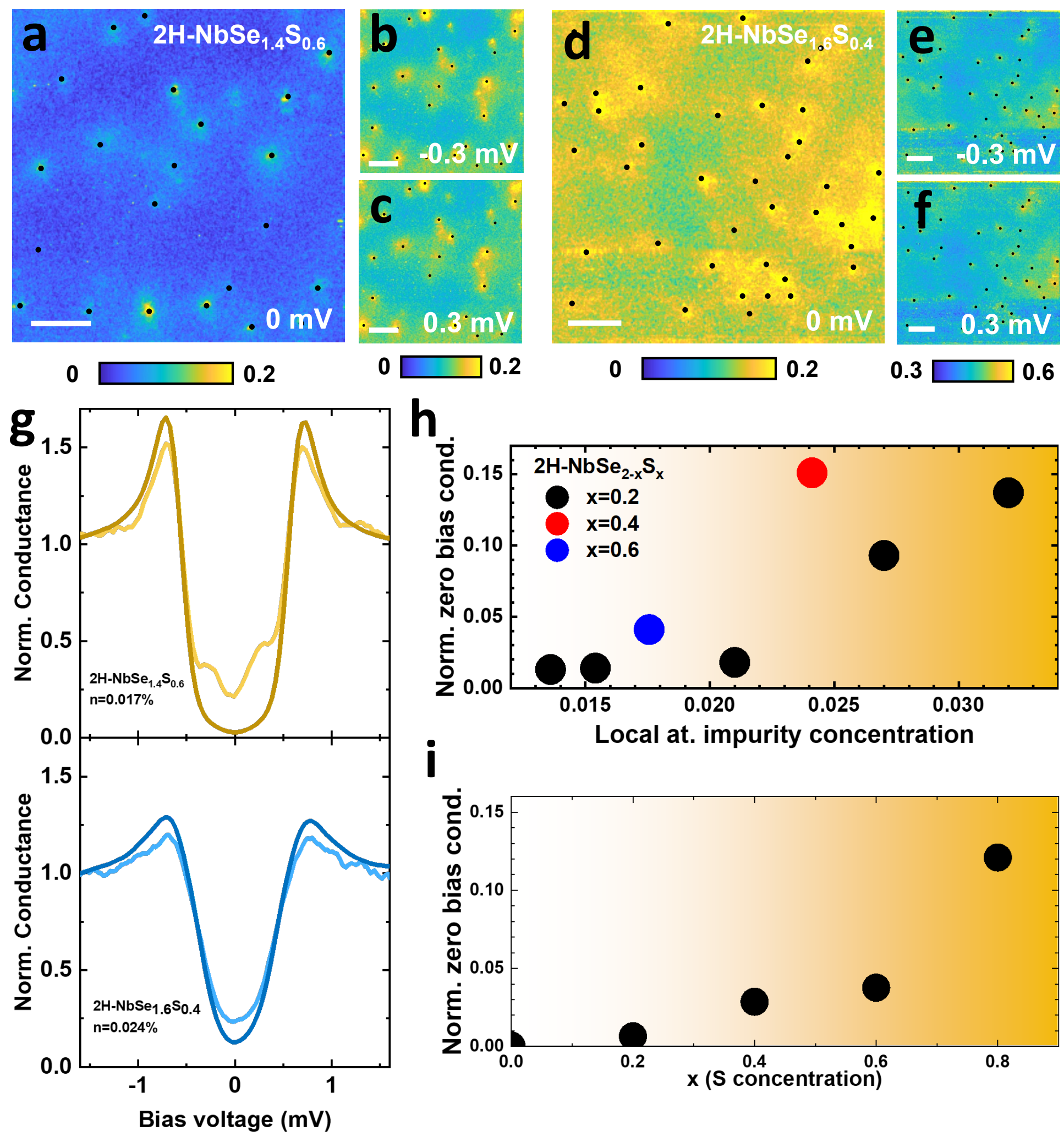

Figure S5 Results in 2H-NbSe$_{1.4}$S$_{0.6}$ and in 2H-NbSe$_{1.6}$S$_{0.4}$. a-c Tunneling conductance maps in 2H-NbSe$_{1.4}$S$_{0.6}$ with $n = 0.017$ at.% magnetic impurity concentration, at zero bias **a** and at ± 0.3 mV **b,c**. Tunneling conductance maps in 2H-NbSe$_{1.6}$S$_{0.4}$, with $n = 0.024$ at.%, at zero bias **d** and at ± 0.3 mV **e,f**. The conductance follows the color scale given at the bottom of the panels. White scale bars are 20 nm long. Black dots mark the position of magnetic impurities. **g** Normalized tunneling conductance curves as a function of the bias voltage obtained on top of a magnetic impurity (light colors) and far from magnetic impurities (dark colors) in 2H-NbSe$_{1.4}$S$_{0.6}$ (bottom panel) and in 2H-NbSe$_{1.6}$S$_{0.4}$ (top panel). **h** Normalized zero bias conductance as a function of the concentration of magnetic impurities n in 2H-NbSe$_{2-x}$S$_x$. **i** Normalized zero-bias conductance for $n \sim 0.02$ at.% as a function of x.

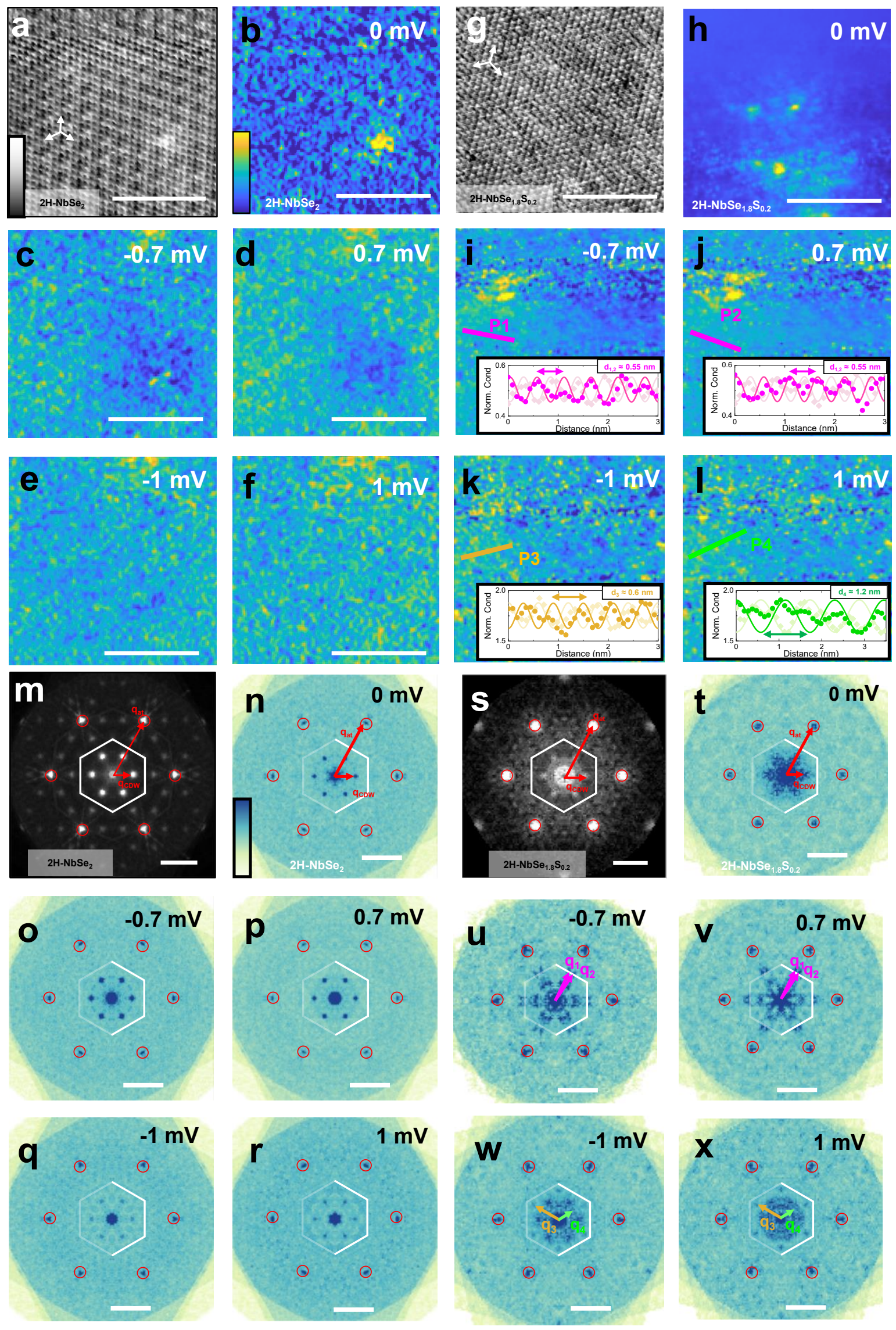

Figure S6 In-gap tunneling conductance around magnetic impurities. In **a,g** we show the STM topography. White scale bars represent 5 nm large in real space and 2 nm^{-1} in reciprocal space. In **b-f** and **h-l** we show the tunneling conductance maps for the bias voltages provided in each panel. Insets in **i-l** provide line scans made along the colored lines as colored points. Light (dark) colored points provide the line scans of the conductance at positive (negative) bias voltage. P_{1-4} stands for for q_{1-4} respectively in Fig.4**g,h,j**. Lines are oscillations with the wave vectors q_{1-4}. In Fig.4**m,s** we provide the Fourier transform of the topographies in **a,g**, respectively. The CDW q_{CDW} and atomic lattice q_{at} Bragg peaks are shown with red arrows. In **n-r** and **t-x** we show the Fourier transform of the tunneling conductance maps from **b-f** and **h-l** at the bias voltages shown in each panel. We mark the $q_1 - q_4$ wave vectors by colored arrows. The white hexagons provide the boundaries of the first Brillouin zone.

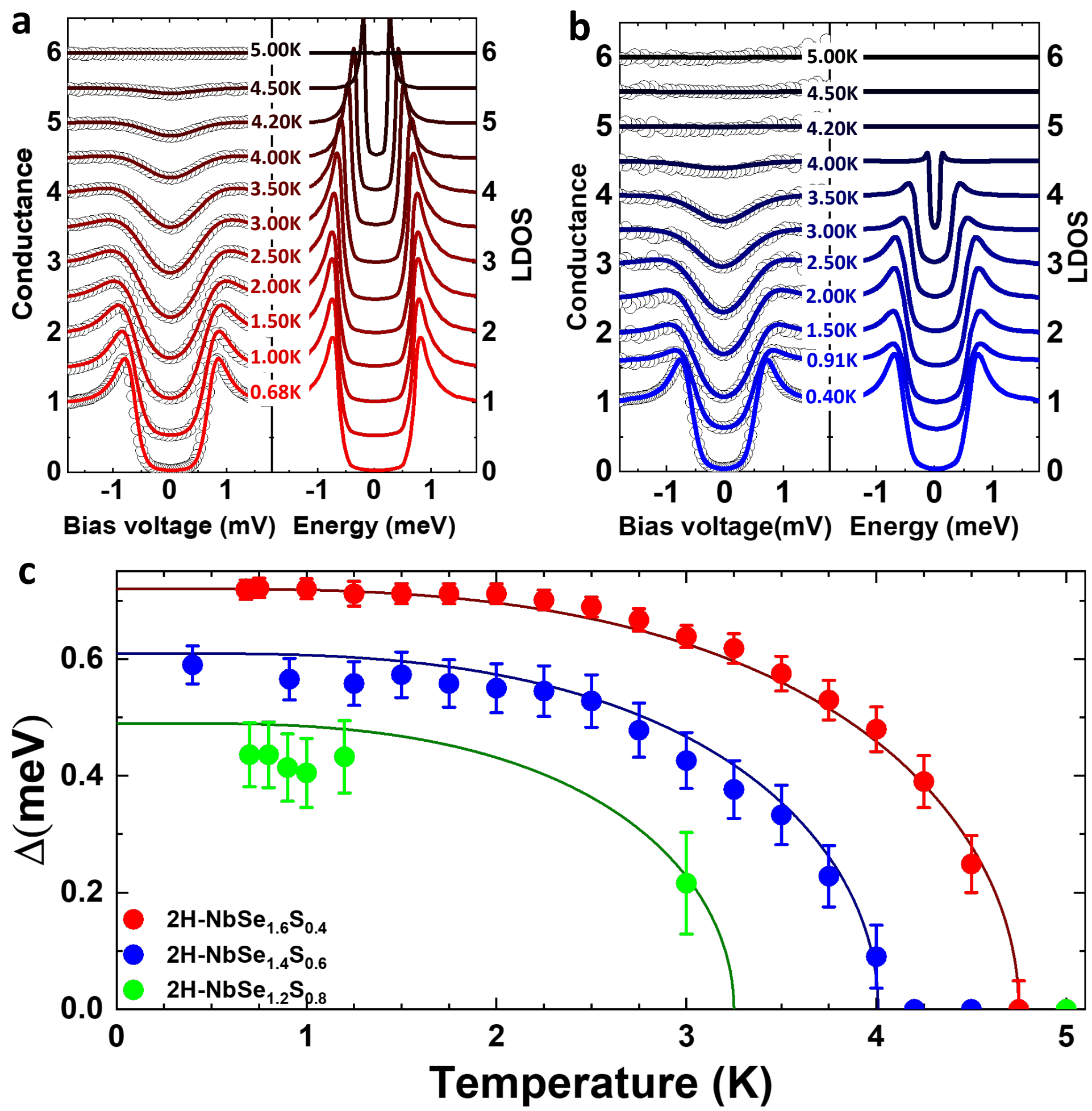

Figure S7 Superconducting density of states as a function of temperature. a,b Tunneling conductance curves vs bias voltage as a function of temperature in 2H-$NbSe_{1.6}S_{0.4}$ and in 2H-$NbSe_{1.4}S_{0.6}$ (in fields of view with the smallest amount of magnetic impurities, less than approximately 0.014 at. %.). Curves are shifted vertically for clarity. Left panels show the tunneling conductance as points and fits as lines. The fits are made at each temperature using the local density of states shown in the right panels. We note that the conductance curves at all measured temperatures exhibit a finite zero-bias conductance, corresponding to a finite density of states at zero energy. At the lowest measured temperature, the zero-bias conductance is comparable to the values shown in Fig.S5**i** for $n \sim 0.02$ at.%. **c** Temperature dependence of the peaks in the density of states. Red dots are for 2H-$NbSe_{1.6}S_{0.4}$, blue dots for 2H-$NbSe_{1.4}S_{0.6}$ and green dots for 2H-$NbSe_{1.2}S_{0.8}$. Solid lines corresponds to the BCS expression using $\Delta = 1.76\, k_B\, T_c$, with $T_c = 4.75\, K$, $T_c = 4\, K$ and $T_c = 3.2\, K$.

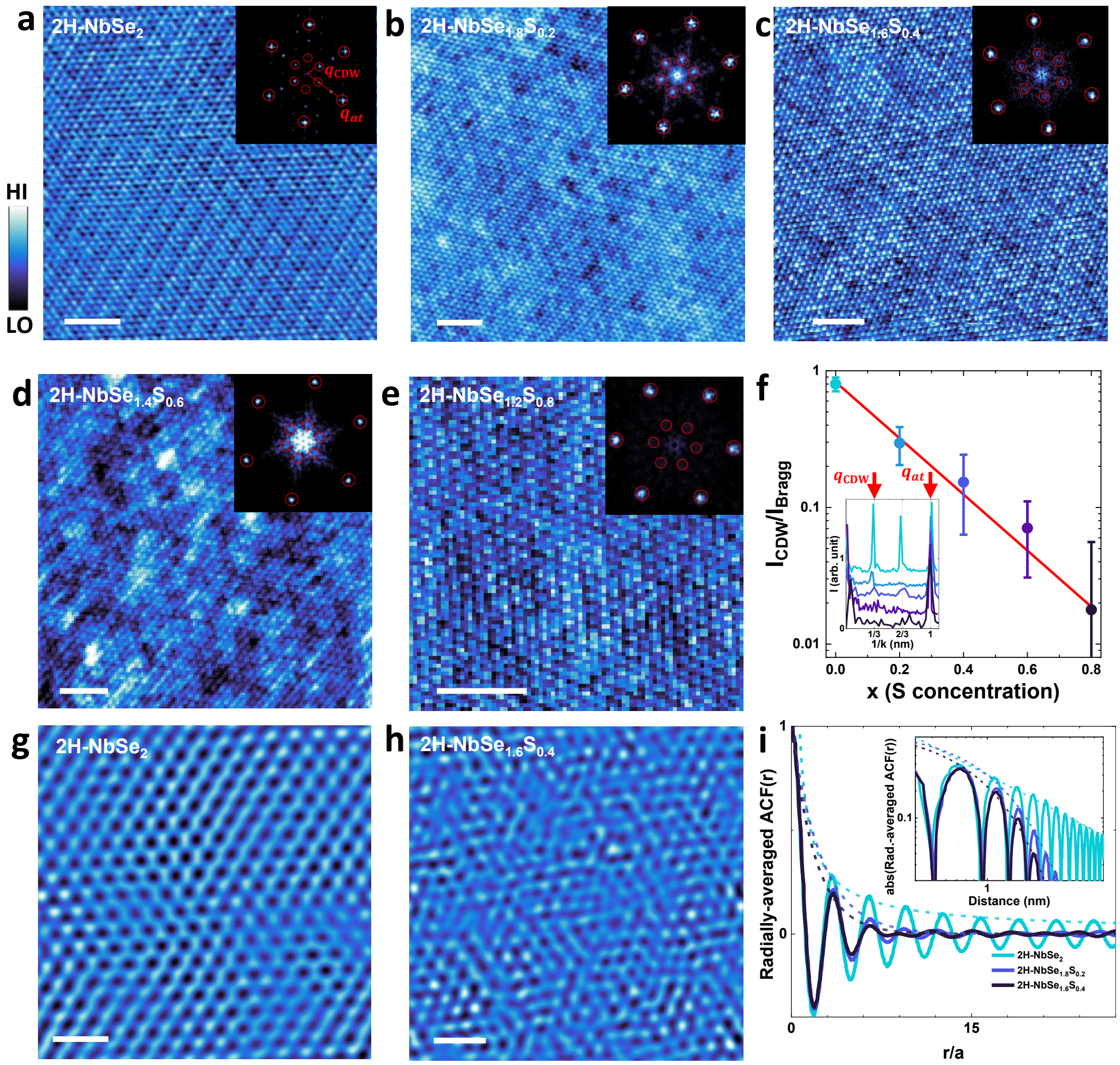

Figure S8 Atomic scale topography of 2H-NbSe$_{2-x}$S$_x$, $0 \leq x \leq 0.8$. **a-e** STM topography images taken in 2H-NbSe$_{2-x}$S$_x$ for $x = 0$ (**a**), $x = 0.2$ (**b**), $x = 0.4$ (**c**), $x = 0.6$ (**d**), $x = 0.8$ (**e**) (bias voltage of 10 mV and current of 0.8 nA). White scale bars represent 3 nm in size. Insets at the top right show the Fourier transform of the images. Red circles show the CDW and atomic Bragg peaks. In **f** we show the ratio of the intensity in the Fourier transform of the CDW peaks and the atomic Bragg peaks. Red line shows an exponential dependence of the peak height with impurity concentration. In the inset we show the profiles along the CDW and Bragg peaks in the FFT maps from $x = 0$ (top curve) to $x = 0.8$ (bottom curve). **g,h** CDW real-space oscillations obtained from filtering the CDW modulation in the topography images **a,c**. (**i**) Radially-averaged autocorrelation function $ACF(r)$, with r being the distance normalized to the ab-plane lattice constant a of the CDW (cyan for 2H-NbSe$_2$, light blue for 2H-NbSe$_{1.8}$S$_{0.2}$ and black for 2H-NbSe$_{1.6}$S$_{0.4}$). Dashed lines show a exponential decay fit to the $ACF(r)$ for the S-doped materials and a power law decay for 2H-NbSe$_2$. In the inset we show the same data as a function of distance in logarithmic scale.

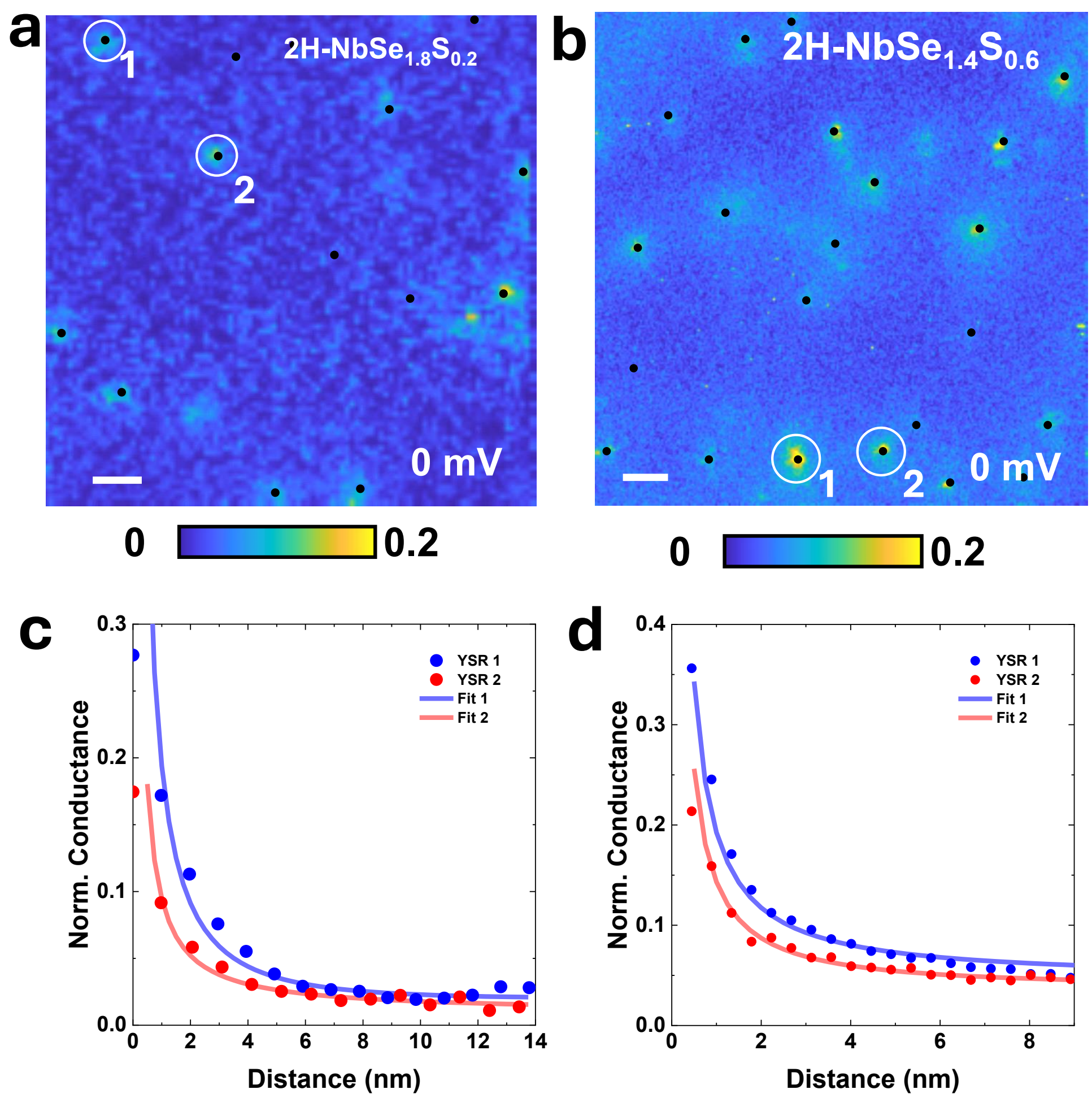

Figure S9 Spatial dependence of the LDOS around magnetic impurities. Zero bias conductance maps in 2H-$NbSe_{1.8}S_{0.2}$ (**a**) and in 2H-$NbSe_{1.4}S_{0.6}$ (**b**). White scale bars represent 10nm. The color scale is shown on the bottom. Magnetic impurities are marked by black dots. **c,d** Radially-averaged tunneling conductance (dots) for the magnetic impurities marked with white circles in **a,b**. Solid lines follow an exponential dependence $\propto e^{-r/r_0}/r + C$.

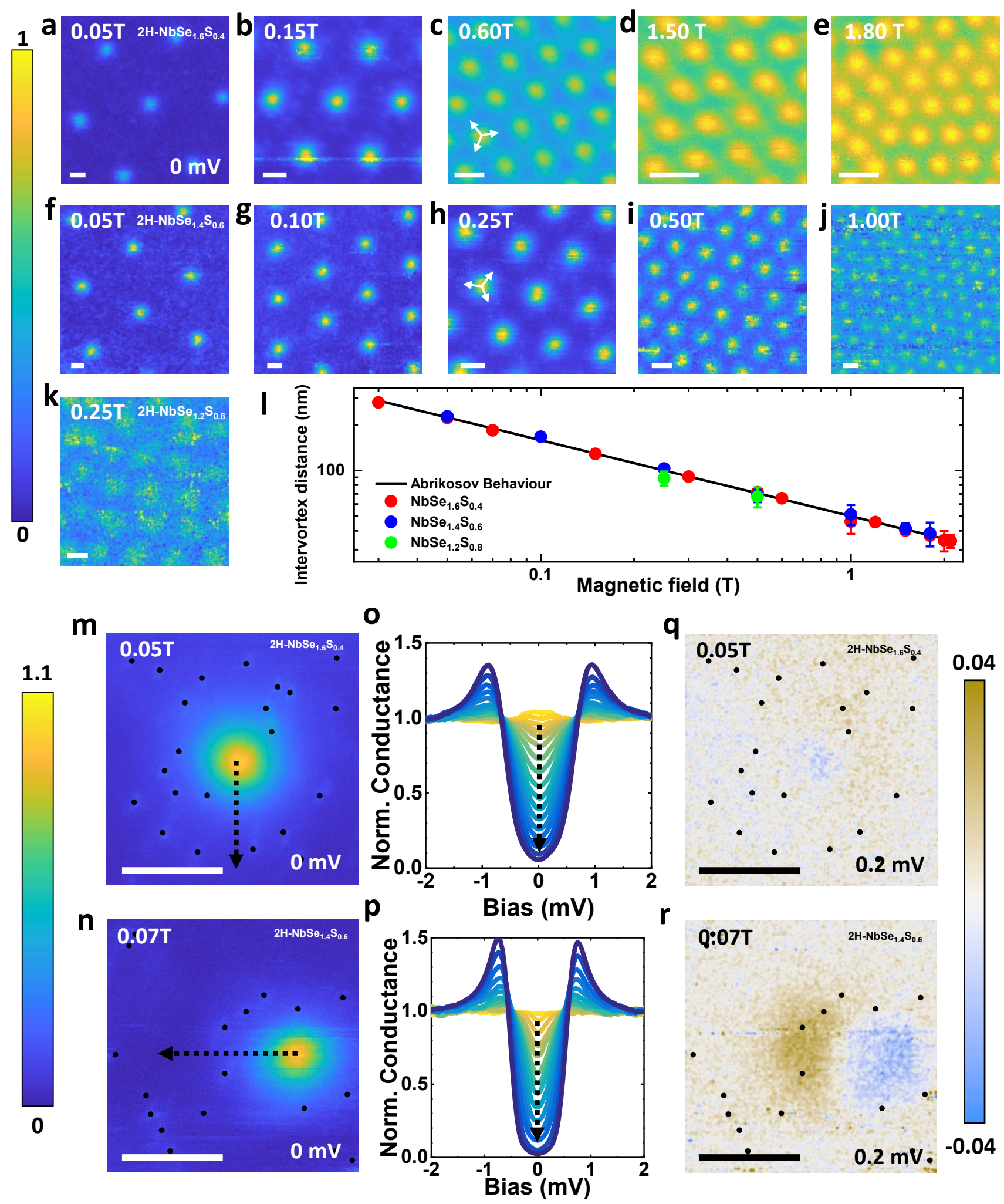

Figure S10 Vortex lattice and vortex core states in 2H-$NbSe_{2-x}S_x$. Tunneling conductance maps at zero bias as a function of the magnetic field in 2H-$NbSe_{1.6}S_{0.4}$ (**a-e**), 2H-$NbSe_{1.4}S_{0.6}$ (**f-j**), and 2H-$NbSe_{1.2}S_{0.8}$ (**k**). Magnetic field values are shown in each panel. The temperature is 400 mK. White arrows indicate the direction of the crystalline directions. The bar at the left provides the color scale in units of the conductance normalized to the value at bias voltages above the gap. Scale bars represent 50 nm. In **l** we show the inter-vortex distance versus magnetic field as red (2H-$NbSe_{1.6}S_{0.4}$), blue (2H-$NbSe_{1.4}S_{0.6}$), and green (2H-$NbSe_{1.2}S_{0.8}$) dots. The black line indicates the Abrikosov prediction for a triangular vortex lattice. In **m,n** we show a zero-bias tunneling conductance map of a single vortex in 2H-$NbSe_{1.6}S_{0.4}$ and in 2H-$NbSe_{1.4}S_{0.6}$. Magnetic fields are marked in each panel. Fe atoms are marked with black dots. In **o,p** we show the tunneling conductance along the dashed black lines in (**m,n**). In **q,r** we show a map obtained by subtracting tunneling conductance maps at positive from negative bias voltages indicated in each panel (± 0.2 mV). The color scale is shown at the right.
